# Endocannabinoid and nitric oxide systems of the hypothalamic paraventricular nucleus mediate effects of NPY on energy expenditure

**DOI:** 10.1016/j.molmet.2018.08.007

**Published:** 2018-09-18

**Authors:** Zoltán Péterfi, Imre Farkas, Raphael G.P. Denis, Erzsébet Farkas, Motokazu Uchigashima, Tamás Füzesi, Masahiko Watanabe, Ronald M. Lechan, Zsolt Liposits, Serge Luquet, Csaba Fekete

**Affiliations:** 1 Department of Endocrine Neurobiology, Institute of Experimental Medicine, Hungarian Academy of Sciences, Budapest, H-1083 Hungary; 2 Unite de Biologie Fonctionnelle et Adaptative, Centre National la Recherche Scientifique, Unité Mixte de Recherche 8251, Université Paris Diderot, Sorbonne Paris Cité, 75205 Paris, France; 3 Multidisciplinary Doctoral School of Sciences and Technology, Budapest, H-1083 Hungary; 4 Department of Anatomy, Hokkaido University School of Medicine, Sapporo 060-8638, Japan; 5 Department of Medicine, Division of Endocrinology, Diabetes and Metabolism, Tupper Research Institute, Tufts Medical Center, Boston, MA, USA; 6 Department of Neuroscience, Tufts University School of Medicine, Boston, MA, USA; 7 Department of Neuroscience, Faculty of Information Technology and Bionics, Pázmány Péter Catholic University, Budapest, H-1083 Hungary

**Keywords:** NPY, Hypothalamic paraventricular nucleus, Endocannabinoid, Nitric oxide

## Abstract

**Objective:**

Neuropeptide Y (NPY) is one of the most potent orexigenic peptides. The hypothalamic paraventricular nucleus (PVN) is a major locus where NPY exerts its effects on energy homeostasis. We investigated how NPY exerts its effect within the PVN.

**Methods:**

Patch clamp electrophysiology and Ca2+ imaging were used to understand the involvement of Ca2+ signaling and retrograde transmitter systems in the mediation of NPY induced effects in the PVN. Immuno-electron microscopy were performed to elucidate the subcellular localization of the elements of nitric oxide (NO) system in the parvocellular PVN. *In vivo* metabolic profiling was performed to understand the role of the endocannabinoid and NO systems of the PVN in the mediation of NPY induced changes of energy homeostasis.

**Results:**

We demonstrated that NPY inhibits synaptic inputs of parvocellular neurons in the PVN by activating endocannabinoid and NO retrograde transmitter systems *via* mobilization of Ca2+ from the endoplasmic reticulum, suggesting that NPY gates the synaptic inputs of parvocellular neurons in the PVN to prevent the influence of non-feeding-related inputs. While intraPVN administered NPY regulates food intake and locomotor activity *via* NO signaling, the endocannabinoid system of the PVN selectively mediates NPY-induced decrease in energy expenditure.

**Conclusion:**

Thus, within the PVN, NPY stimulates the release of endocannabinoids and NO *via* Ca^2+^-influx from the endoplasmic reticulum. Both transmitter systems appear to have unique roles in the mediation of the NPY-induced regulation of energy homeostasis, suggesting that NPY regulates food intake, energy expenditure, and locomotor activity through different neuronal networks of this nucleus.

## Highlights

• NPY increases the intracellular Ca^2+^ level of PVN neurons by mobilizing the Ca^2+^ from ER.
• NPY inhibits the input of these neurons by endocannabinoids and NO.
• IntraPVN administered NPY regulates food intake and locomotor activity *via* NO signaling.
• IntraPVN administered NPY regulates energy expenditure *via* the endocannabinoid system.
• NPY regulates the energy expenditure and food intake *via* different neuronal networks of the PVN.

## Graphical abstract

**Figure:**
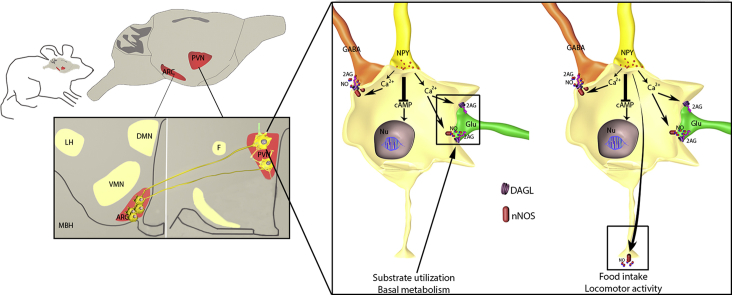

## 1 Introduction

Neuropeptide Y (NPY) is a key peptide in the central regulation of energy homeostasis [1]. Central administration of NPY markedly stimulates food intake and inhibits energy expenditure [2]. The NPY-synthesizing neuronal population that has the most important role in the regulation of energy homeostasis is located in the ventromedial part of the hypothalamic arcuate nucleus. Synthesis of NPY and the second orexigenic peptide produced by these cells, Agouti related peptide (AgRP), are stimulated by fasting and inhibited by administration of leptin [1]. These neurons, referred as NPY/AgRP neurons, project to second order feeding-related neuronal groups and transmit the information derived from peripheral signals toward these feeding centers. One of the main brain regions where NPY exerts its effects on food intake and energy expenditure is the hypothalamic paraventricular nucleus (PVN). NPY-containing axons of arcuate nucleus origin densely innervate the PVN [3], and form functional inhibitory synapses on parvocellular PVN neurons [3], [4]. Fasting increases the NPY secretion in the PVN [5]. In addition, focal administration of NPY into the PVN markedly increases food intake [6] and carbohydrate utilization [7], decreases energy expenditure, the expression of uncoupling protein 1 in brown adipose tissue [8], [9], and induces body weight gain [10].

The effects of NPY on its target cells are exerted *via* three G protein-coupled receptors, Y1, Y2, and Y5 receptors [11]. While Y1 and Y5 receptors are present on postsynaptic neurons, the Y2 receptor is primarily presynaptic and regulates neurotransmitter release [11]. Both postsynaptic NPY receptors are expressed in the PVN [12], coupled to pertussis-toxin sensitive G_i/o_ proteins [13], and lead to the inhibition of cAMP accumulation by inhibiting adenylate cyclase [11]. Thus, some of the effects of NPY on energy expenditure are exerted through the regulation of gene expression in the PVN *via* the modulation of the cAMP pathway [14], [15], [16], [17]. NPY also exerts rapid effects on the electrophysiological properties of the parvocellular neurons of the PVN. It inhibits the firing of the parvocellular neurons and also inhibits the GABAergic inputs of these cells [18], [19]. However, it is unclear whether NPY inhibits the GABAergic inputs *via* presynaptic receptors or *via* retrograde messengers released from the neurons in the PVN.

Since activation of the Y1 receptor causes an increase in intracellular Ca^2+^ in cultured neuroblasts and human pulmonary arterial smooth muscle cells [20], [21], and the increase in intracellular Ca^2+^ concentration is known to activate the synthesis of retrograde transmitters [22], [23], we hypothesized that retrograde transmitters, such as endocannabinoids and nitric oxide (NO), may be involved in the NPY-induced inhibition of presynaptic terminals and in the mediation of the effects of NPY on the energy homeostasis. To test this hypothesis, we determined the role of the endocannabinoid and NO systems in the mediation of the effects of NPY within the PVN *in vitro* and *in vivo*.

## 2 Results

### 2.1 Regulation of the synaptic inputs of parvocellular neurons in the PVN by NPY involves release of Ca^2+^ from the endoplasmic reticulum and activation of retrograde transmitter systems

**Figure 1:**
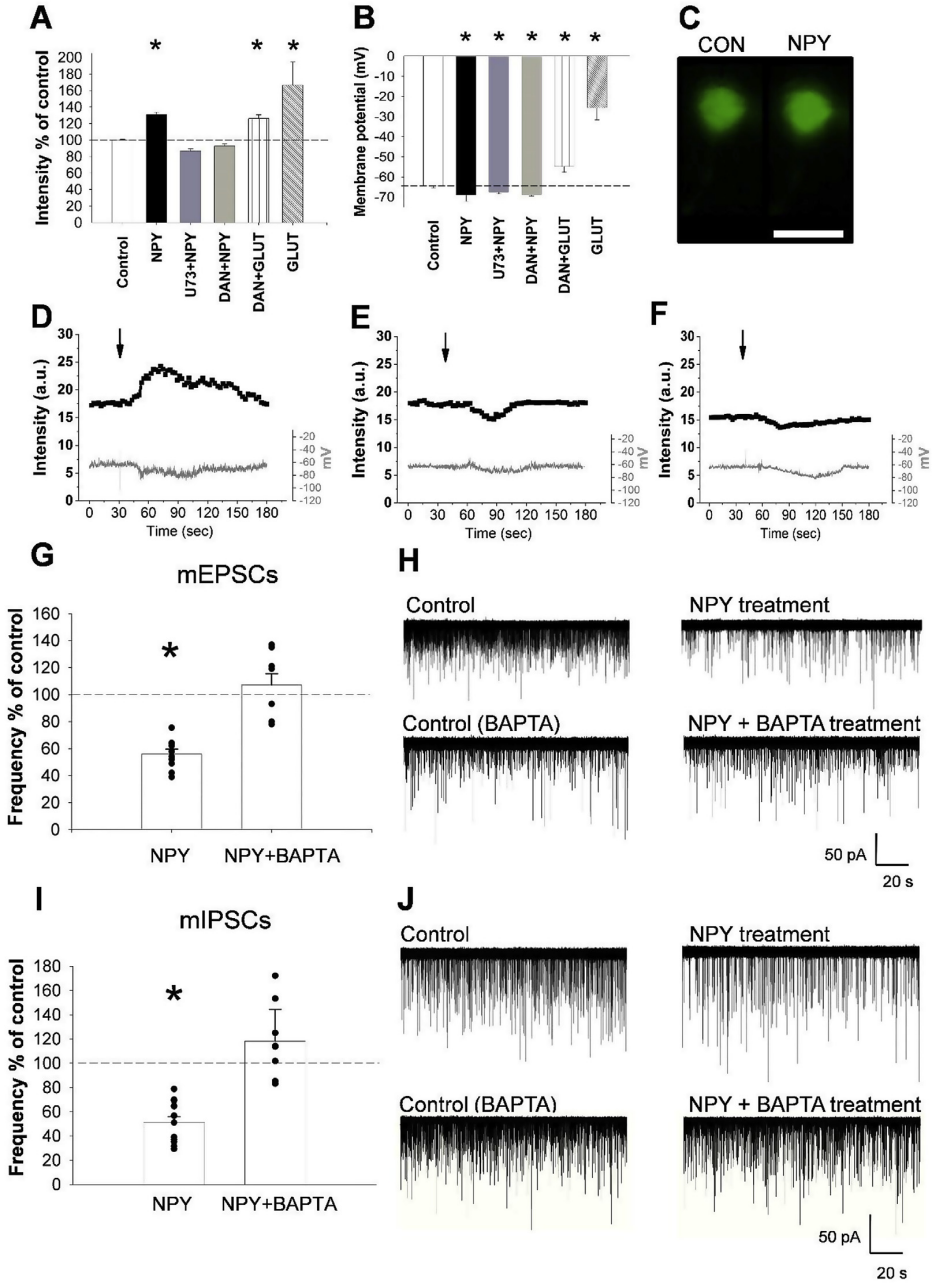
**NPY-induced inhibition of the synaptic input of parvocellular neurons in the PVN requires increase of intracellular Ca**^**2+**^**levels and retrograde transmitters**. Bar graphs (A, B) illustrate the changes of fluorescent intensity (A) and membrane potential (B) of OGB-1 loaded parvocellular PVN neurons in response to NPY alone (N_cells_ = 9; N_mice_ = 6) or NPY in the presence of PLCβ inhibitor U73122 (U73; 5 μM; N_cells_ = 8; N_mice_ = 6) or the specific ryanodine receptor inhibitor Dantrolene (DAN; 5 μM; N_cells_ = 17; N_mice_ = 13). Glutamate treatment (A, B) was used as positive control (N_cells_ = 8; N_mice_ = 6). While the NPY induced increase of intracellular Ca^2+^ levels were prevented by both antagonists, the effect of glutamate was not prevented by Dantrolene (DAN + GLUT, N_cells_ = 7; N_mice_ = 6). The NPY-induced hyperpolarization was not influenced by the antagonists. Fluorescent images compare the fluorescent intensity of an OGB-1 loaded parvocellular neuron before (CON) and after (NPY) the NPY treatment (C). Representative traces illustrate the effects of NPY alone (D) and in the presence U73122 (E) or Dantrolene (F) on the fluorescent intensity (upper plot) and membrane potential (lower plot) of OGB-1 loaded parvocellular neurons. Arrows indicate the beginning of the NPY treatment. The effect of NPY and NPY + BAPTA treatment on the event frequencies of mEPSCs (G, H) and mIPSCs (I, J) in acute slice preparation using whole-cell patch-clamp recordings. Bar graphs (G, I) show that NPY (1 μM) treatment caused a strong decrease in frequency of events in both mEPSCs (N_cells_ = 10; N_mice_ = 6) and mIPSCs (N_cells_ = 12; N_mice_ = 5), while pretreatment of the cells with BAPTA (10 mM) completely abolished this effects of NPY in both mEPSCs (N_cells_ = 8; N_mice_ = 6) and mIPSCs (N = 9; N_mice_ = 5). (H, J) display representative recordings of different treatment groups. Bars represent mean ± SEM; *P < 0.05, [F(4,44) = 29.075) according to the ANOVA analysis followed by SNK post hoc test. Scale bar = 20 μm on panel (C).

To understand the effects of NPY on the parvocellular neurons of the PVN, patch clamp electrophysiology combined with calcium imaging were performed. NPY treatment induced a robust increase of the fluorescence intensity of the calcium sensitive OGB-1 (131.05 ± 2.58%, N_cells_ = 9, N_mice_ = 6, P < 0.001, T = −10.325) in parvocellular neurons and caused hyperpolarization of these cells (control *vs.* NPY treated (mV): −64.35 ± 1.91 *vs.* −69.02 ± 3.24) (Figure 1A–D).

Since NPY increases the intracellular Ca^2+^ levels *via* mobilization of Ca^2+^ from the endoplasmic reticulum in neuroblastoma cells [21], and activation of phospholipase C-beta (PLCβ) is known to mobilize Ca^2+^ from this cell compartment [24], we tested whether the NPY-induced increase of intracellular Ca^2+^ is mediated by this pathway. The PLCβ inhibitor, U73122 (5 μM), completely prevented the NPY-induced increase of OGB-1 fluorescence in parvocellular neurons in the PVN (86.73 ± 2.52%, N_cells_ = 8, N_mice_ = 6, P = 0.45; Figure 1A,B,E) without influencing the effects of NPY on the membrane potential (control *vs.* NPY treated (mV): −64.63 ± 1.71 *vs.* −67.62 ± 0.89, N_cells_ = 8, N_mice_ = 6; P = 0.003, T = 3.319; Figure 1B,E). Dantrolene (5 μM), a specific ryanodine receptor inhibitor that blocks the Ca^2+^ release from the endoplasmic reticulum, also completely blocked the NPY-induced increase of intracellular Ca^2+^ (92.68 ± 2.56%, N_cells_ = 17, N_mice_ = 13; P = 0.66; Figure 1A,F) without preventing NPY-induced hyperpolarization (control *vs.* NPY treated (mV): −64.28 ± 0.66 *vs.* −68.93 ± 0.62, N_cells_ = 17, N_mice_ = 13; P < 0.0001, T = 4.493; Figure 1B,F). In accordance with data in the literature showing that the effect of glutamate on the intracellular Ca^2+^ level is at least partly independent from mobilization of Ca^2+^ from the endoplasmic reticulum [25], glutamate (100 μM) could still increase the OGB-1 fluorescence intensity (151.01 ± 10.19%, N_cells_ = 8; N_mice_ = 6, P = 0.01, T = −4.454; Figure 1A) and depolarize the neurons in the presence of Dantrolene (control *vs.* glutamate (mV): −65.27 ± 0.89 *vs.* −22.18 ± 4.20, N_cells_ = 7; N_mice_ = 6, P < 0.0001, T = 3.821; Figure 1B). These data indicate that the NPY-induced increase of intracellular Ca^2+^ level is mediated by PLCβ acting on the endoplasmic reticulum, and the effect of NPY on membrane potential is independent from this process.

To test the effects of NPY on the presynaptic inputs of parvocellular neurons in the PVN, the miniature excitatory and inhibitory postsynaptic currents (mEPSC and mIPSC) of the parvocellular neurons were studied. NPY significantly decreased the event frequencies of both the mEPSCs (55.3 ± 3.0% of control, N_cells_ = 19, N_mice_ = 11; P = 0.009, T = 2.926; Figure 1G,H) and the mIPSCs (51.2 ± 4.7% of control; N_cells_ = 13, N_mice_ = 6; P < 0.001, T = 4.426; Figure 1I,J). This treatment had no effect on other measured parameters of miniature currents including the peak amplitude (Supplementary Figure 1A,B), time to peak, half-width, rise time and rise tau. The NPY-induced decrease in event frequencies without change of peak amplitude suggested that NPY inhibited presynaptic terminals either directly *via* presynaptic receptors or indirectly by releasing retrograde transmitters from the parvocellular neurons.

To differentiate between these two possibilities, the calcium chelator BAPTA was added to the intracellular solution to block calcium signaling only in the studied neuron. The presence of BAPTA completely prevented the NPY-induced decrease of both mEPSC (107.0 ± 8.4% of its control, N_cells_ = 8, N_mice_ = 6; P = 0.43) and mIPSC (118.0 ± 9.74% of its control, N_cells_ = 9, N_mice_ = 5 P = 0.56) event frequencies (Figure 1G,I, Supplementary Figures 2A, 3A). The event frequencies of the cells treated with both BAPTA and NPY were significantly different from that of cells treated with NPY alone (mEPSCs P = 0.003, T = -4.427; mIPSCs P < 0.001, T = −5.554; Figure 1G,I). These data excluded the possibility that NPY acts directly on the presynaptic terminals of parvocellular neurons, and demonstrated the importance of increased intracellular Ca^2+^ levels in the mediation of the NPY-induced effects. Moreover, these data demonstrate that NPY regulates the presynaptic inputs of the parvocellular neurons of the PVN *via* retrograde transmitters.

### 2.2 Endocannabinoid and nitric oxide systems are anatomically positioned to regulate both the excitatory and inhibitory synaptic inputs of parvocellular neurons in the PVN

**Figure 2:**
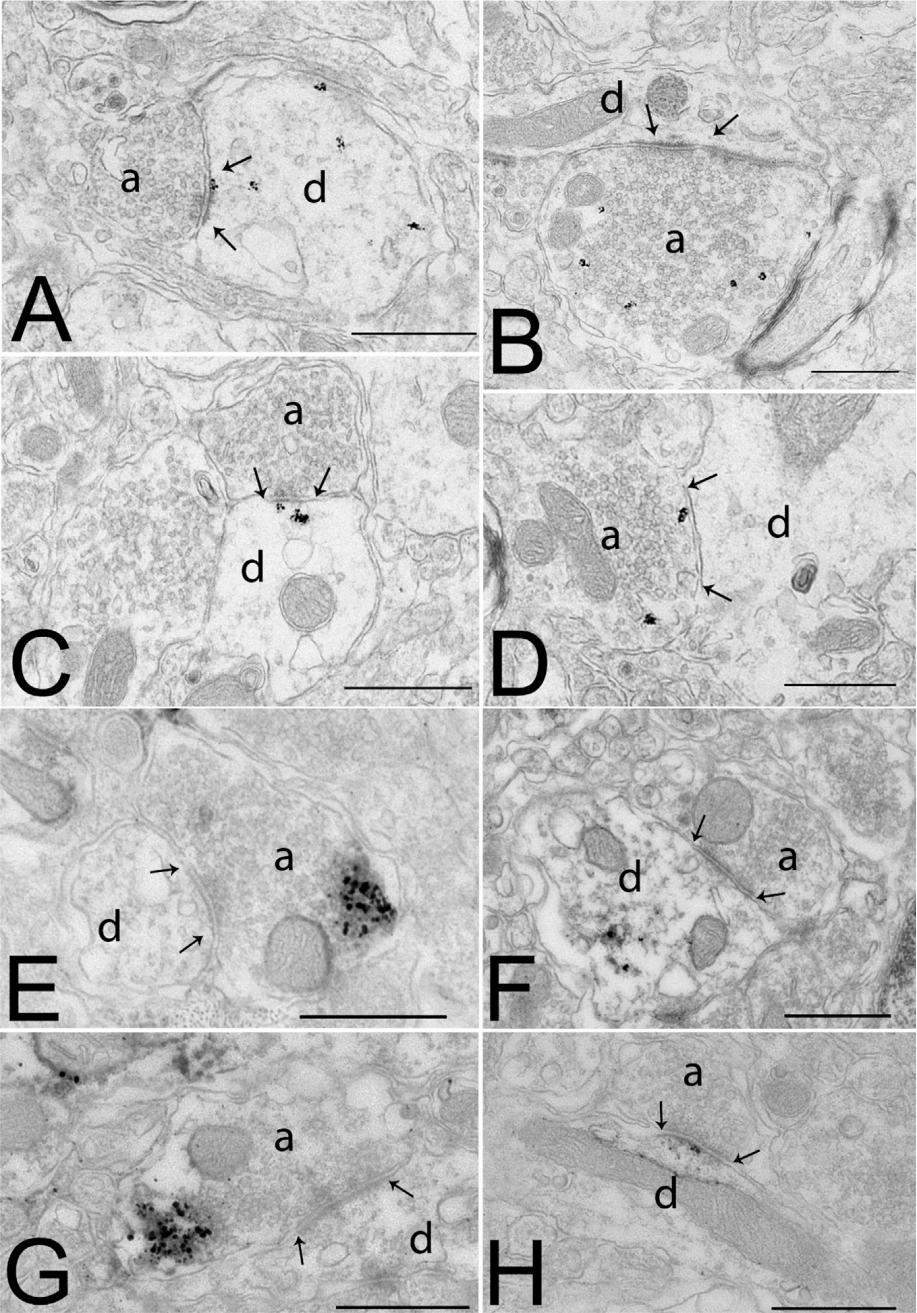
**Ultrastuctural localization of the components of the nitric oxide system in the parvocellular part of the hypothalamic paraventricular nucleus (PVN) in mice**. Electron micrographs illustrate the postsynaptic (A, C) and presynaptic (B, D) localization of nNOS-immunoreactivity in the parvocellular part of the PVN. The nNOS-immunoreactivity is labeled with highly electron dense gold–silver particles and observed in the proximity of both asymmetric (A, B) and symmetric (C, D) synapses. Soluble guanylyl-cyclase α1 subunit-immunoreactivity (sGCα1-IR) is present both in presynaptic axons (E, G) and in dendrites (F, H) in the paraventricular nucleus. sGCα1-immunoreactivity is recognized by the presence of the electron dense silver grains. sGCα1-immunoreactivity is present in axon varicosities forming both symmetric (E) and asymmetric type synapses (G). sGCα1-immunoreactivity is also seen in dendrites in the proximity of the postsynaptic density of both symmetric (F) and asymmetric (H) synapses. Arrows point to synapses. Abbreviations: a = axon; d = dendrite. Scale bars = 0.5 μm.

Previously, we demonstrated the presence of the CB1 receptor in both inhibitory and excitatory inputs of parvocellular neurons of the mouse PVN [26]. To determine whether NO can also function as a retrograde transmitter in the parvocellular PVN, ultrastructural studies were performed. The neuronal nitric oxide synthase (nNOS) enzyme, the primary neuronal isoform of the NOS family [27], was observed in neuronal perikarya and dendrites in addition to presynaptic terminals in the parvocellular part of the PVN (Figure 2A–D). In many instances, silver grains denoting nNOS-immunoreactivity were closely associated with the postsynaptic density of both symmetric (Figure 2C) and asymmetric type synapses (Figure 2A). The α1 subunit of soluble guanylate cyclase (sGC), the primary receptor for NO, was also observed in presynaptic terminals and postsynaptic elements of both excitatory and inhibitory synapses (Figure 2E–H). These data demonstrate that NO may function as both an anterograde and retrograde transmitter in the parvocellular part of the PVN.

### 2.3 Anatomical relationship of the endocannabinoid and nitric oxide systems in the PVN

**Figure 3:**
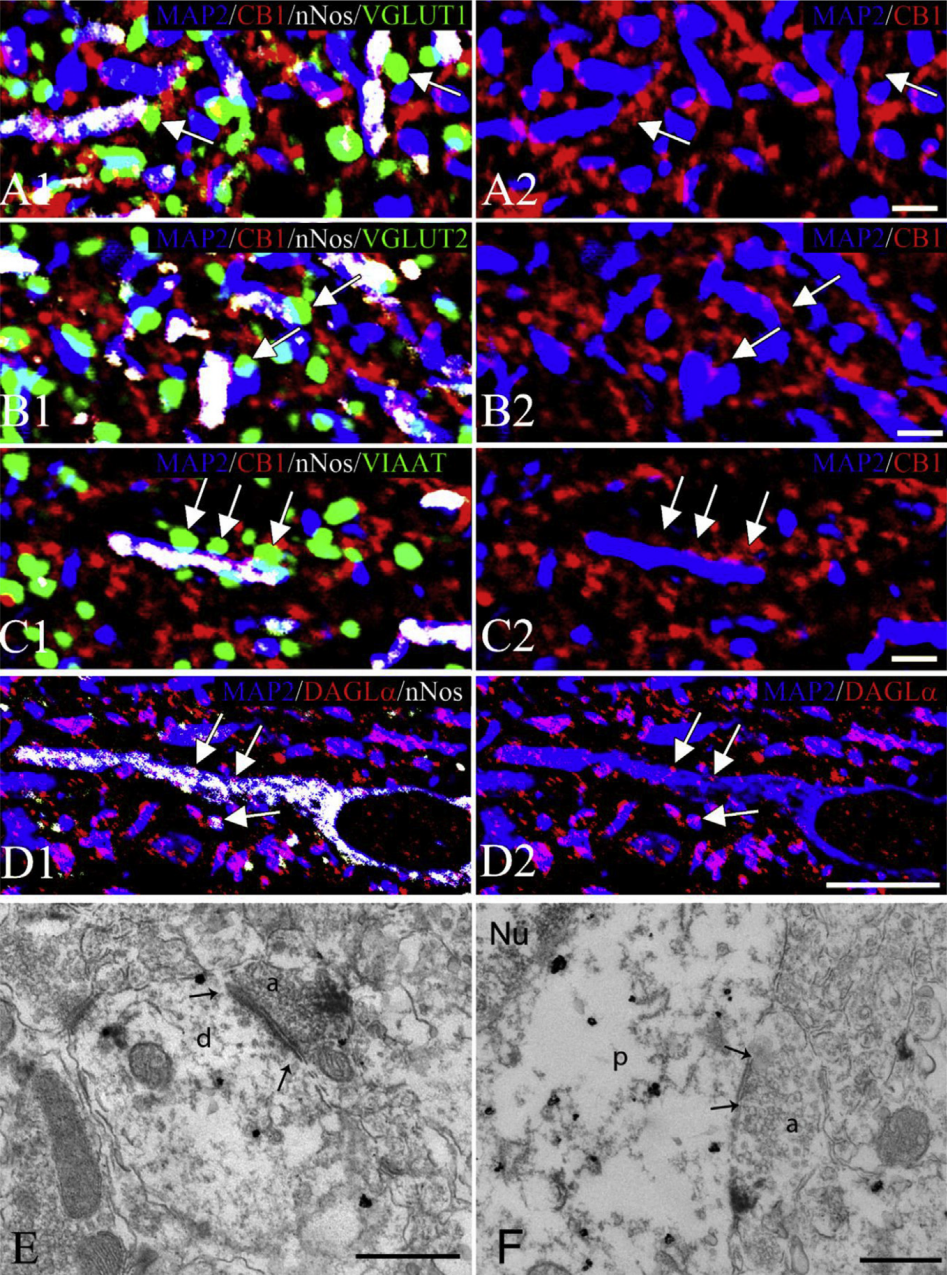
**Association of the endocannabinoid and nitric oxide (NO) systems with the same synapses of parvocellular neurons in the hypothalamic paraventricular nucleus (PVN)**. Images of quadruple immunolabeled preparations (A–C) illustrate the close association of CB1-immunoreactive (IR) excitatory (A, B) and inhibitory (C) axon varicosities with nNOS containing dendrites in the parvocellular part of the PVN. The axon varicosities of excitatory neurons are labeled with vesicular glutamate transporter 1 (VGLUT1)- (A, green) or vesicular transporter 2 (VGLUT2)- (green, B) immunoreactivity, while the inhibitory terminals are labeled with vesicular inhibitory amino acid transporter (VIAAT)-immunoreactivity (green, C). The dendrites are visualized based on their MAP2-immunoreactivity (blue). CB1-immunoreactivity is labeled with red fluorescence, while the nNOS-immunoreactivity is pseudocolored white. For better visualization, the MAP2 and CB1-immunoreactivities in the same field are also shown separately (A2-C2). Images of triple-labeled immunofluorescent preparations (D) show the colocalization (arrows) of the endocannabinoid synthesizing diacylglycerol lipase alpha (DAGLα; red) and nNOS (white) in the dendrites (MAP2-IR, blue) of the parvocellular part of the PVN. Electron micrographs illustrate synaptic associations (arrows) between a nNOS-IR dendrite (E) or perikaryon (F) with CB1-IR axon terminals. The nNOS-IR elements are labeled with highly electron dense gold–silver granules, while the CB1-IR terminals are recognized by the presence of the electron dense Ni-DAB chromogen. The associations of the two retrograde transmitter systems can be observed in both asymmetric (E) and symmetric (F) type of synapses in the parvocellular part of the PVN. Arrows point to the synapses. Scale bars = 2 μm on (A–C), 10 μm on (D), and 0.5 μm on (E, F). Abbreviations: a = axon; d = dendrite; Nu = nucleus; p = perikaryon.

To determine whether the endocannabinoid and NO systems regulate the very same neuronal inputs of parvocellular neurons in the PVN, quadruple-labeling immunocytochemistry was performed using a MAP2 neuronal marker combined with markers of glutamatergic or GABAergic terminals and components of the endocannabinoid and NO systems. In many instances, nNOS was present in dendrites of parvocellular neurons in the PVN close to the region where CB1-IR glutamatergic (VGLUT1- or VGLUT2-IR; Figure 3A,B) or GABAergic (VIAAT-IR; Figure 3C) terminals formed juxtapositions with dendrites. In addition, the endocannabinoid 2-AG synthesizing enzyme diacylglycerol lipase alpha (DAGLα) was observed to colocalize with nNOS in dendrites of parvocellular neurons (Figure 3D). To further support the anatomical basis of an interaction between the two retrograde transmitter systems, ultrastructural studies were performed, showing that nNOS is present in close proximity to the postsynaptic site of the synapse of some CB1-IR glutamatergic and GABAergic terminals (Figure 3E,F). These data suggest that the two, retrograde, transmitter systems may interact to regulate the inputs of parvocellular neurons.

### 2.4 Both the endocannabinoid and the nitric oxide systems are involved in the mediation of the inhibitory effects of NPY on synaptic inputs of parvocellular neurons in the PVN

**Figure 4:**
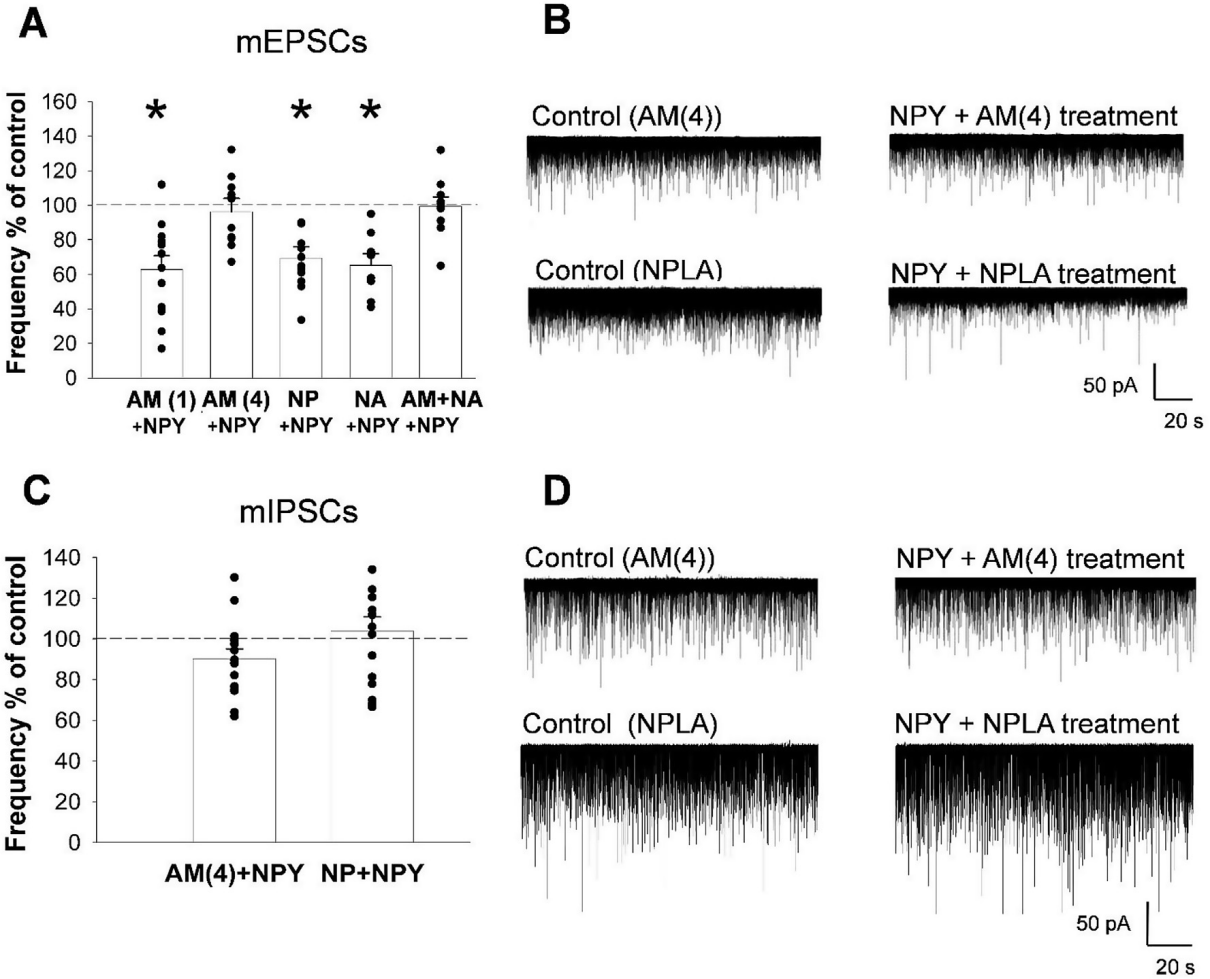
**Involvement of the endocannabinoid and nitric oxide (NO) systems in the mediation of the effects of NPY on the synaptic inputs of parvocellular neurons of the hypothalamic paraventricular nucleus (PVN)**. Bar graphs (A, C) and representative whole-cell patch-clamp recordings (B, D, and Supplementary Figure 4C) of mEPSCs (A, B, Supplementary Figure 4C) and mIPSCs (C, D) illustrate the effects of CB1 and nNOS inhibition on the synaptic input of parvocellular neurons in the PVN. CB1 receptor antagonist inhibits NPY-induced reduction of event frequencies of mEPSCs in a dose dependent manner (A). Administration of 1 μM CB1 receptor antagonist AM251 failed to influence the effect of NPY (1 μM) on the mEPSCs (N_cells_ = 12; N_mice_ = 6), however, 4 μM AM251 blocked the NPY-induced inhibition of mEPSCs frequencies (A, B; N_cells_ = 12; N_mice_ = 4). The higher dose of AM251 also prevented the NPY-induced changes of mIPSCs frequencies (C, D; N_cells_ = 15; N_mice_ = 6). Neither the selective nNOS inhibitor NPLA (100 nM; N_cells_ = 11; N_mice_ = 7), nor the non-selective NOS inhibitor l-NAME (100 μM; N_cells_ = 8; N_mice_ = 6), influenced the effect of NPY on the mEPSC frequencies (A, B). In contrast, the same dose of NPLA abolished the effect of NPY on the event frequencies of mIPSCs (C, D; N_cells_ = 15; N_mice_ = 6). Combination of l-NAME and the subthreshold dose of AM251 (1 μM), however, abolished the effect of NPY on the mEPSCs (A; N_cells_ = 10; N_mice_ = 6). Bars represent mean ± SEM; *P < 0.05 [F(5,68) = 10.641] according to the ANOVA analysis followed by SNK post hoc test. Abbreviations: AM(1): 1 μM AM251 and NPY treatment; AM(4): 4 μM AM251 and NPY treatment; NP: NPLA and NPY treatment; NA: l-NAME and NPY treatment; AM + NA: 1 μM AM 251 and l-NAME (100 μM) co-treatment with NPY.

To test the involvement of the endocannabinoid system in mediating the actions of NPY on the input of parvocellular neurons, the effect of NPY treatment was tested on mPSCs of parvocellular neurons in the presence of the CB1 receptor antagonist, AM251. Treatment of slices with AM251 (4 μM) abolished the effect of NPY on mEPSCs, thus event frequency of mEPSCs did not decrease significantly in response to NPY (91.0 ± 8.2% of its control, N_cells_ = 12, N_mice_ = 4 P = 0.19; Figure 4A,B, Supplementary Figure 2C). Treatment group comparison (ANOVA for mEPSC frequency values) indicated a significant inhibition of NPY induced effects (P < 0.001, [F(5,68) = 10.641]) in mEPSCs event frequencies (Figure 4A). Administration of AM251 (4 μM) also markedly decreased the NPY-induced decrease of mIPSCs (Figure 4C,D, Supplementary Figure 3B). No significant difference was observed between NPY-treatment and baseline periods in the presence of the CB1 antagonist (90.19 ± 4.88% of control; N_cells_ = 15, N_mice_ = 6; P = 0.07). The AM251 treatment alone had no effect on the frequency of mPSCs (Supplementary Figures 2B,C, 3B, 4A,B)

To test the involvement of the NO system, the effect of the non-selective NOS inhibitor, l-NAME (100 μM), and the selective nNOS inhibitor, NPLA (100 nM), was studied. Treatment of slices with these inhibitors alone did not modified the mPSC frequencies (Supplementary Figures 2D,F, 3C, 4A,B). Neither l-NAME nor NPLA influenced the effects of NPY on mEPSC frequencies (65.3 ± 5.29% of its control; N_cells_ = 8, N_mice_ = 6; P < 0.001, T = 6.494 and 69.36 ± 6.63% of its control, N_cells_ = 11, N_mice_ = 7; P = 0.004, T = 3.124, respectively; Figure 4A,B, Supplementary Figures 2D,F, 4C). Selective inhibition of nNOS (NPLA; 100 nM), however, prevented the effect of NPY on the mIPSC event frequency (103.8 ± 7.0% of its control; N_cells_ = 15, N_mice_ = 6, P = 0.749; Figure 4C,D and Supplementary Figure 3C). The combined NPLA + NPY treatment abolished the effect of NPY on the frequency of mIPSCs; supporting an important role of NO in NPY-induced regulation of the inhibitory inputs of parvocellular neurons (Figure 4C,D and Supplementary Figure 3C).

Inhibition of NO signaling, alone, did not influence the effect of NPY on the excitatory inputs of parvocellular PVN neurons. Since elements of the NO signaling pathway were present in excitatory synapses, we tested whether the NO and endocannabinoid systems interact in these excitatory synapses. To do so, a subthreshold dose of AM251 (1 μM) was combined with inhibitors of NOS. At this concentration, 1 μM, AM251 had no effect on the NPY-induced decrease in event frequencies of mEPSCs (62.7 ± 8.0% of its control, N_cells_ = 12, N_mice_ = 6; P = 0.0004, T = 4.811; Figure 4A and Supplementary Figures 2B, 4C). Co-administration of 1 μM AM251 with l-NAME (100 μM), however, completely blocked the inhibitory effect of NPY on mEPSC event frequencies (99.3 ± 5.5% of its control, N_cells_ = 10, N_mice_ = 6; P = 0.90; Figure 4A and Supplementary Figures 2E, 4C) when compared to baseline values.

### 2.5 Endocannabinoid and the NO systems mediate different functions of NPY in the PVN in the regulation of energy homeostasis

**Figure 5:**
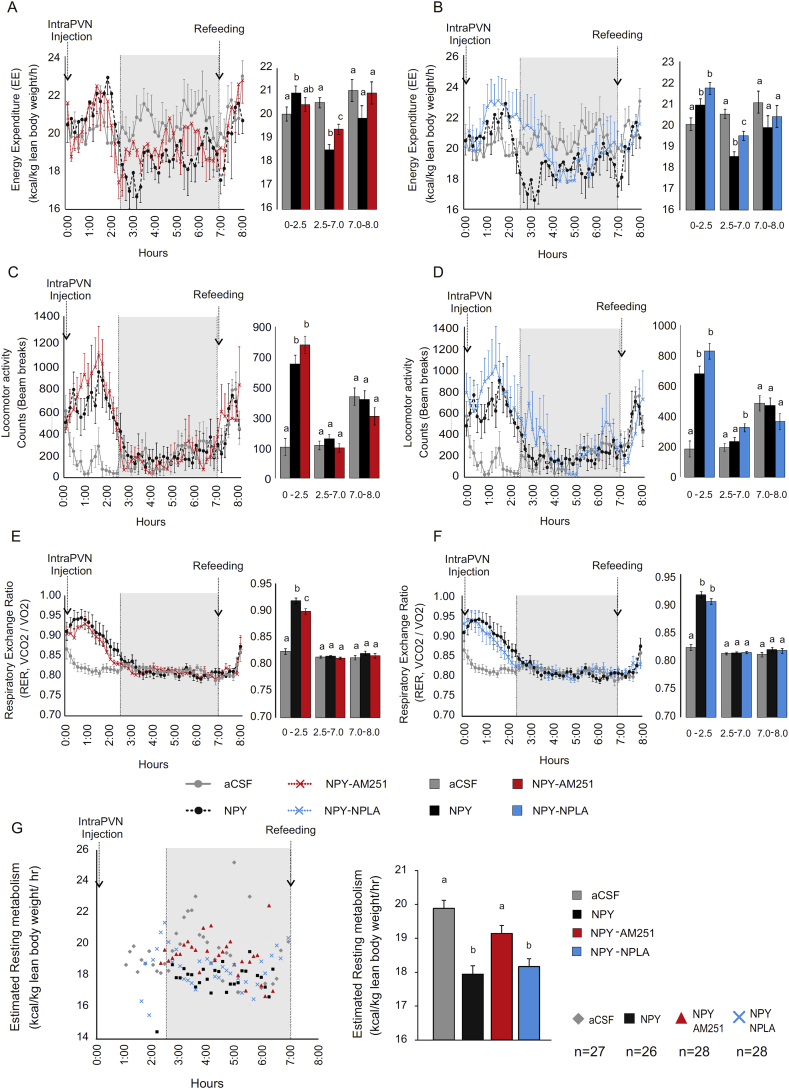
**Effect of intraPVN co-administration of NPY with the CB1 antagonist, AM251, or the nNOS inhibitor, NPLA, on parameters of energy expenditure**. Effect of intraPVN co-administration of NPY and AM251 on energy expenditure normalized by total lean body mass (A), spontaneous locomotor activity (C), and respiratory exchange ratio (RER) (E) of n = 8 mice with no access to food. While NPY significantly decreased the energy expenditure in the second period (2.5 h–7h), AM251 attenuated this inhibitory effect of NPY (A). Inhibition of endocannabinoid signaling did not influence the effect of NPY on the locomotor activity (C) and had only minor effect on the NPY induced increase of RER (E) in the first period (0h–2.5 h). Effect of intraPVN co-administration of NPY and NPLA on energy expenditure normalized by total lean body mass (B), spontaneous locomotor activity (D), and RER (F) of n = 7 mice with no access to food. Inhibition of NO signaling attenuated the NPY induced inhibition of energy expenditure at the beginning of second period (B). This effect of nNOS inhibitor overlapped with the nNOS inhibition induced prolongation of the stimulatory effect of NPY on the locomotor activity (D). Inhibition of NO signaling had no effect on the RER (F). Estimation of the resting metabolism of mice injected with NPY alone or co-administrated with AM251 or NPLA (G), as described in Supplementary Methods, 27, 26, 28 and 28 points were collected respectively for CSF, NPY, NPY-AM251 and NPY-NPLA treated groups within the second phase (between 2.5 h and 7 h, grey area). NPY markedly inhibited the resting energy expenditure. This effect was attenuated by AM251, but was not influenced by NPLA. Each group was injected either with CSF (grey), NPY (black), NPY + AM251 (red), NPY + NPLA (blue) at 0 h, and food was replaced at 7 h. Bar graphs were calculated from the ANOVA analysis for each phase. Data with different superscript letters are significantly different (P < 0.05) according to the ANOVA analysis followed by a Bonferroni *post hoc* test. Data are expressed as mean ± SEM.

To determine the role of the two retrograde transmitter systems in mediating the various actions of NPY on energy balance within the PVN, we studied whether local administration of inhibitors of the endocannabinoid and NO systems influence the effects of NPY on food intake, energy expenditure, substrate utilization, and locomotor activity. NPY administration into the PVN (intraPVN) impacted on whole energy expenditure, spontaneous activity, and respiratory exchange ratio (RER), with a characteristic biphasic pattern if the animals had no access to food (Figure 5A–F; black lines and bars), in good agreement with previous observations [28], [29], [30]. As the biphasic response is prototypical of the NPY-initiated response, the two phases were independently analyzed. Therefore, the experiments were split into two main periods, 0–2.5 h and 2.5–7 h, after which food was given to the mice at 7 h.

The first 2.5 h after central intraPVN NPY injection was characterized by a slight increase in energy expenditure (F = 3.04, T = 2.463, NPY *vs*. aCSF, P < 0.05; Figure 5A,B; black lines and bars), primarily attributable to increased locomotor activity (F = 35.87, T = 7.13, NPY *vs*. aCSF, P < 0.001; Figure 5C,D; black lines and bars) as ANCOVA with locomotor activity as a covariate revealed there was a strong effect of locomotor activity on the energy expenditure (F = 31.04, T = −5.572, P < 0.001). In this period, an NPY induced increase of carbohydrate utilization was indicated by the increase of RER (F = 113.6, T = 14.48, NPY *vs*. aCSF, P < 0.001; Figure 5E,F; black lines and bars). The second phase post injection (2.5 h–7h) was characterized by a significant decrease in energy expenditure (F = 27.58, T = -7.396, NPY *vs*. aCSF P < 0.001. Figure 5A,B; black lines and bars) and normalization of both locomotor activity and substrate utilization compared to aCSF-treated animals (Figure 5C,D,E,F; black lines and bars). These changes persisted until food was reintroduced to the animals at 7 h.

Fasting is typically associated with sustained increase in NPY release from arcuate nucleus neurons to PVN structures and associated with a transient increase in foraging and hyperphagic behavior (after an overnight fast) followed by a drastic decrease in activity as a means to conserve energy [31], [32]. A similar change in locomotor activity also occurs in food restricted animals before the time of anticipated feeding when the NPY release is increased in the PVN [33], [34]. IntraPVN NPY administration seems to recapitulate these behavior outputs in the presence or absence of food (Supplementary Figure 5).

Local administration of AM251 into the PVN had no effect on the energy expenditure and the locomotor activity (Supplementary Figure 6). AM251 only slightly increased the RER in the first period without influencing it in the second period (F = 18.86, T = -6.046, CSF *vs*. AM251 P < 0.001, Supplementary Figure 6). Local administration of NPLA alone had no effect on any of the measured parameters (Supplementary Figure 6). Local administration of NPLA before intraPVN NPY treatment, however, resulted in an extended period of increased locomotor activity (F = 10,34 T = 3.95: CSF *vs*. NPY-nPLA, P < 0.001, Figure 5D). Antagonizing the CB1 signaling pathway through local AM251 administration did not oppose NPY action on locomotor activity, either in the first or second period (Figure 5C), but resulted in a significant change in substrate utilization as evidenced by attenuation of the NPY-induced increase in RER during the first hyperactive period after NPY administration (F = 68.77 T = -3.312, P < 0.01 NPY *vs*. NPY-AM251, Figure 5E). NPLA administration, however, did not affect RER in either period (Figure 5F).

Importantly, between 2.5 h and 7 h, both AM251 and NPLA treatments partially reversed the decrease in energy expenditure induced by NPY (F = 27.58, T = 3.104 and F = 19.70, T = 2,863 respectively for NPY-AM251 and NPY-nPLA *vs*. NPY P < 0.01. Figure 5A,B). Though, the action of NPLA on the NPY-mediated inhibition of energy expenditure overlapped with the period of increased locomotor activity suggesting that it could be attributed to the extended hyperactivity (Figure 5D). In contrast, the effect of AM251 on the energy expenditure was not associated with a change in locomotor activity (Figure 5C), suggesting that AM251 attenuates the effect of intraPVN NPY on the resting energy expenditure. Therefore, an estimation of the resting energy expenditure, defined by a steady resting condition, absence of locomotor activity and no access to food (as described in supplemental section), was calculated during the 2.5–7 h period, showing that while AM251 markedly attenuated the inhibitory effect of NPY on resting energy expenditure, NPLA treatment had no effect on this parameter (F = 13.98: T = −5.657: CSF *vs*. NPY P < 0.001, T = −5.104: CSF *vs*. NPY-nPLA P < 0.001, T = 3.528: P < 0.01 NPY *vs*. NPY-AM251 Figure 5G).

**Figure 6:**
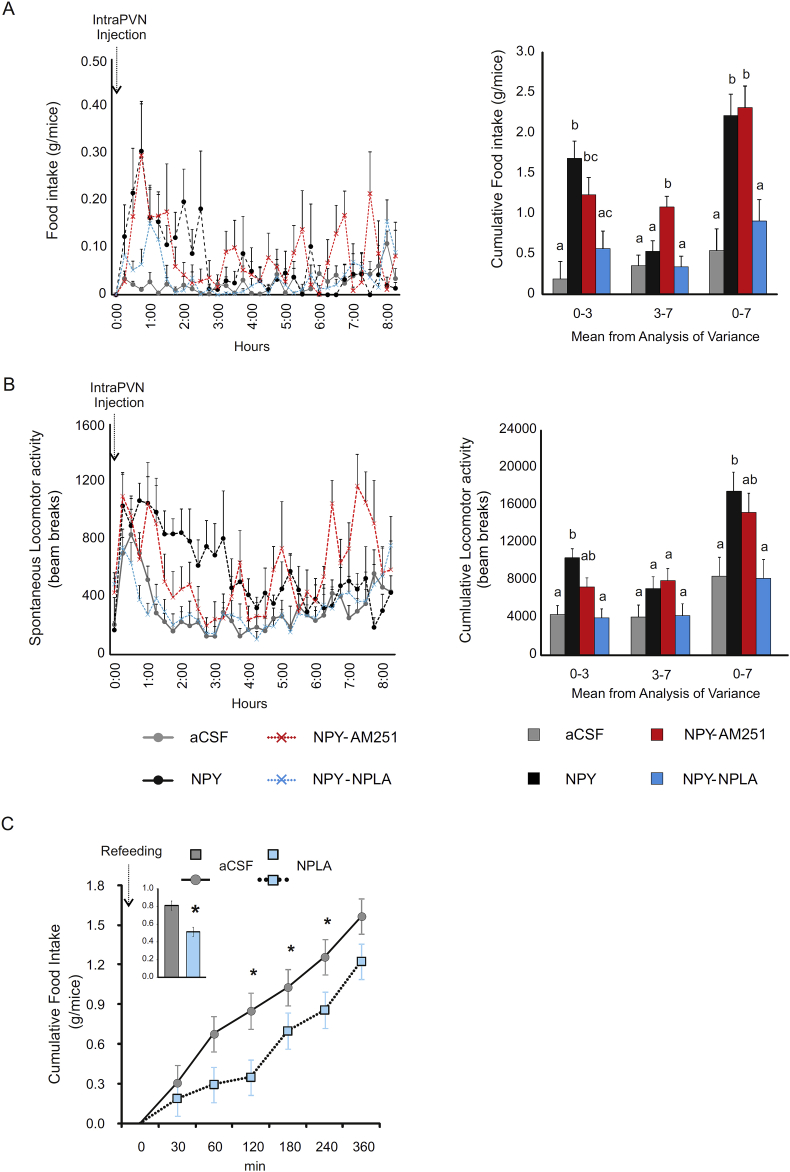
**Effect of intraPVN co-administration of NPY with the CB1 antagonist, AM251, or the nNOS inhibitor, NPLA, on food intake and locomotor activity.** Food intake (A) and spontaneous activity (B) of mice injected intraPVN with CSF (grey), NPY alone (black) and NPY co-administrated with AM251 (red) or NPLA (blue). (See also in Supplementary Figure 7) Food intake and spontaneous locomotor activity was monitored over 8 h post injection. The cumulative graph is presented in Supplementary Figure 7. While AM251 did not influenced the cumulative food intake of NPY treated mice, NPLA prevented the NPY induced increase of food intake (A). Similarly, AM251 had only minor effect on the locomotor activity, while NPLA completely prevented the NPY induced increase of the locomotor activity in the presence of food (B). IntraPVN injection of NPLA alone (light blue) diminished food intake following an overnight fast (C). Mean ± SEM extracted from the analysis of variance of food intake and spontaneous locomotor activity are represented in the bar graphs. Data with different superscripts letters are significantly different (P < 0.05) according to the ANOVA followed by Bonferroni post hoc test. Data are expressed as mean ± SEM. n = 8 mice per group.

In a separate set of experiments, the involvement of the two retrograde signaling pathways was investigated in the mediation of the effect of acute intraPVN administration of NPY on the food intake. Analysis of food intake and spontaneous locomotor activity was split into two phase, 0–3 h and 3–7 h, in accordance with the observed feeding pattern (Figure 6A). IntraPVN NPY administration resulted in a more than 5-fold increase in food intake (F = 9.61, T = 4.902, CSF *vs*. NPY P < 0.001) and 2-fold increase in locomotor activity (F = 9.52, T = 4.422, CSF *vs*.NPY P < 0.001) during the first 3 h (Figure 6A,B).

IntraPVN administration of the endocannabinoid antagonist AM251 alone, compared to aCSF, or in combination with NPY, compared to NPY, had only a marginal effect on food intake and locomotor activity (Figure 6A,B, Supplementary Figure 7A,C). In contrast, the inhibition of nNOS signaling within the PVN completely prevented the NPY-induced increase in food intake (F = 9.61, T = -3.666, NPY-nPLA *vs*. NPY P < 0.01) and locomotor activity (F = 9.52 T = −4.681 NPY-nPLA *vs*. NPY P < 0.001) (Figure 6A,B, Supplementary Figure 7B,D), while administration of NPLA, alone, had no effect on either parameter (Supplementary Figure 7B,D).

As exogenously administered NPY increased the NPY concentration in the PVN before food was provided in our experimental paradigm, and fasted animals also have increased NPY concentration in the PVN before they receive food, we tested whether inhibition of NO signaling within the PVN can influence the behavior of fasted and refed mice. Similar to intraPVN injection of NPY, overnight fasted mice showed voracious feeding when food was reintroduced and had increased locomotor activity (Figure 6C, Supplementary Figure 8A,B). IntraPVN administration of the nNOS inhibitor, NPLA, markedly decreased the food intake (F = 16.89 T = −4.110, aCSF *vs*. nPLA P < 0.001, Figure 6C) but not the locomotor activity (Supplementary Figure 8A), suggesting that the endogenously released NPY utilizes NO to influence these parameters (Figure 6C) conditioned by the energetic status of the animals.

## 3 Discussion

NPY is one of the most potent orexigenic molecules known, and its effects on energy expenditure and food intake are at least in part mediated by neurons in the PVN [1]. During fasting, when the hunger drive is increased, there is increased NPY release in the PVN, and administration of NPY directly into this nucleus of sated rats markedly increases food intake and decreases energy expenditure [35]. However, little information is available about how NPY exerts these effects on energy homeostasis within the PVN. In this study, we demonstrate that in addition to the well-known effects of NPY on gene expression of parvocellular PVN neurons [14], [15], a rapid and direct effect of NPY occurs by gating neuronal inputs to these cells *via* activation of retrograde signaling systems.

Using patch clamp electrophysiology, we have shown that, in agreement with a previous report [36], NPY hyperpolarizes parvocellular neurons in the PVN, suggesting that NPY rapidly inhibits the electric activity of these neurons. At the same time, however, NPY increases intracellular Ca^2+^ level in parvocellular cells by mobilizing Ca^2+^ from the endoplasmic reticulum. The effect of NPY on intracellular Ca^2+^ may be cell type specific [13]. According to the literature [13], in neurons, NPY may inhibit L-type Ca^2+^ channels by inhibiting cAMP accumulation, or may inhibit presynaptic N- and P/Q type voltage gated Ca^2+^ channels, thus it can prevent the increase of intracellular Ca^2+^level induced by other transmitters. Similar to our results, however, NPY can also increase intracellular Ca^2+^ level as demonstrated in smooth muscle cells and neuroblasts [20], [21]. Our data demonstrate that, in parvocellular neurons of the PVN, the effect of NPY is mediated through PLCβ and the resultant release of Ca^2+^ from the endoplasmic reticulum. It is likely that this effect of NPY is induced by activation of Gβγ subunit rather than G_i/o_α subunit-mediated inhibition of cAMP [13]. Our data do not exclude the possibility that NPY also inhibits voltage sensitive Ca^2+^ channels in the parvocellular neurons and thus, prevents the intracellular increase of Ca^2+^ induced by activation of other receptors.

Earlier studies demonstrated that NPY inhibits the mIPSCs of the parvocellular neurons in the PVN of rats without influencing the mEPSCs [18], [37]. In mice, however, we demonstrate NPY-induced inhibition of both the mIPSCs and mEPSC in parvocellular neurons of the PVN. The explanation for the different observations may be the different dose of NPY used in the studies, or the species differences. The inhibitory effects of NPY on the mPSCs could be exerted directly on presynaptic terminals or *via* retrograde transmitter release from the parvocellular neurons. However, our observation that intracellular administration of the Ca^2+^ chelator, BAPTA, completely prevented the influence of NPY on the inputs of parvocellular neurons excluded the possibility that NPY acts directly on the presynaptic terminals innervating the parvocellular neurons.

Previously, our laboratories showed that the endocannabinoid retrograde signaling system is present in the PVN by demonstrating that CB1 is localized in both excitatory and inhibitory presynaptic axon varicosities innervating parvocellular PVN neurons [26]. In addition, we showed that the endocannabinoid system is essential for mediating the inhibitory action of ghrelin on the excitatory inputs of these cells [38]. Similar to the endocannabinoid system, the NO system is also known to regulate energy homeostasis [39], and in the hippocampus, it is utilized as both an anterograde and retrograde transmitter [40]. The role of NO as a retrograde transmitter in the PVN, however, had not been previously described.

In this study, we provide both light microscopic and ultrastructural data to establish that both nNOS and the major NO receptor, SGc, are present in both pre- and postsynaptic sites within the parvocellular subdivision of the PVN, indicating that the NO system could be utilized as both an anterograde and retrograde transmitter in the PVN. Furthermore, we observed that in a population of the inputs of parvocellular PVN neurons, elements of both the NO and endocannabinoid retrograde signaling systems are present, indicating that the two signaling systems may interact to regulate synaptic inputs to PVN neurons.

Using patch clamp electrophysiology, we found that both retrograde signaling mechanisms are indeed involved in the mediation of the effects of NPY on the parvocellular neurons. Inhibition of CB1 prevented the effects of NPY on mEPSCs, indicating that activation of endocannabinoid signaling is a necessary step in the mechanism by which NPY inhibits the excitatory input to the PVN. Inhibition of NO synthesis, alone, had no effect, but the combination of a subthreshold dose of a CB1 antagonist together with a nNOS inhibitor was able to prevent the effect of NPY on mEPSCs suggesting that activation of NO signaling can facilitate the actions of NPY. In contrast, NPY-induced inhibition of the inhibitory inputs of the parvocellular neurons was prevented by inhibition of both retrograde signaling systems, even when these inhibitors were administered alone, before the NPY treatment. This suggests that both retrograde signaling systems together are necessary for the NPY- induced inhibition of inhibitory inputs.

In many synapses, retrograde signaling molecules have an important role in homosynaptic feedback, i.e. in response to synaptic activity, postsynaptic neurons release retrograde transmitters to inhibit their active presynaptic terminals [41]. However, this mechanism does not appear to be involved in the interaction of arcuate nucleus NPY neurons and parvocellular neurons of the PVN, as NPY neurons do not express CB1 receptors [42]. Furthermore, NPY release remains increased over the course of fasting in the PVN [35]. These observations suggest that NPY gates non-feeding related inputs of parvocellular PVN neurons by the release of endocannabinoids and NO, preventing any overriding effects of synaptic inputs in the PVN that may impede fasting-induced regulation of parvocellular neurons that play such a critical role in the regulation of energy homeostasis.

*In vivo* inhibition of nNOS within the PVN completely prevented the potent stimulatory effect of the intraPVN administration of NPY on food intake. Furthermore, inhibition of nNOS decreased food intake even if NPY was not administered exogenously, but the endogenous NPY release within the PVN was increased in fasted animals before food was reintroduced. Antagonizing nNOS with NPLA prolonged the stimulatory effects of NPY on locomotor activity without influencing the RER. The NPY-induced parallel change of RER and locomotor activity, suggested that the NPY-induced increase in carbohydrate utilization was induced by increased locomotor activity and not by a direct, central effect of NPY. However, NPLA treatment only prolonged the NPY-induced increase in locomotor activity without influencing substrate utilization, indicating that the effects of NPY on locomotor activity and substrate utilization are mediated *via* separate mechanisms within the PVN.

In contrast, antagonizing the endocannabinoid signaling system in the PVN had no effect on food intake, as demonstrated by the absence of a response when a CB1 antagonist was administered simultaneously with NPY directly into the PVN, or during food restriction. Nevertheless, endocannabinoid signaling would appear to be involved in mediating the NPY-induced effect on energy expenditure, as inhibition of CB1, in contrast to nNOS, prevented ∼50% of the NPY-induced decrease in energy expenditure. This observation is similar to the effect of NPY on glutamatergic synapses, which can be blocked by AM251 but not NPLA. Therefore, we hypothesize that NPY inhibits energy expenditure *via* inhibition of the excitatory inputs of the parvocellular neurons.

In contrast to the endocannabinoid system, NO is utilized as both a retrograde and anterograde transmitter in the PVN. While the nature of patch clamp methodology allowed the examination of NO as retrograde transmitter, *in vivo* inhibition of nNOS in the PVN blocked both anterograde and retrograde NO transmission. Since all of the retrograde transmitter-mediated effects of NPY could be prevented in patch clamp studies with administration of the CB1 antagonist, AM251, but the *in vivo* effects of NPY on the food intake and locomotor activity was influenced only by local inhibition of NO signaling within the PVN, we hypothesize that the effects of NPY on food intake and locomotor activity are mediated by local neuronal circuits utilizing NO as an anterograde transmitter.

Intriguingly, inhibition of NO synthesis in the PVN had different effects on NPY-induced locomotor activity in the presence or absence of food. When food was not available, NPLA prolonged the NPY-induced increase in locomotor activity, while in the presence of food, NPLA completely blocked NPY-induced locomotor activity. Therefore, it is likely that NPY stimulates locomotor activity *via* different mechanisms in the presence or absence of food.

However, the main source of the NPY innervation of the PVN is the arcuate nucleus, catecholaminergic NPY neurons of the brainstem also innervate the parvocellular neurons of the PVN [15], [43]. Thus activation of these inputs may also gate the synaptic inputs of the PVN neurons, but currently less information is available about the role of these brainstem inputs.

In summary, NPY seems to have a rapid action on parvocellular PVN neurons by gating both inhibitory and excitatory inputs to these neurons *via* activation of endocannabinoid and NO retrograde signaling systems. The *in vivo* studies demonstrate that both transmitter systems have critical roles in mediating the effects of NPY on food intake, energy expenditure, and locomotion. However, the differential effects of endocannabinoids and NO on the different aspects of NPY signaling in the PVN indicates that NPY exerts its effects on the different parameters of energy hormostasis *via* separate neuronal circuits within the PVN.

## 4 Methods

### 4.1 Animals

The experiments were carried out on adult, male, CD1 mice (from local breeding colony of IEM HAS or from Janvier-SAS, France), weighing 30–35 g, housed under standard environmental conditions (light between 06:00 h and 18:00 h, temperature 22 ± 1 °C, mouse chow, and water *ad libitum*). All experimental protocols were reviewed and approved by the Animal Welfare Committee at the Institute of Experimental Medicine of the Hungarian Academy of Sciences, the Animal Care Committee of the University Paris Diderot-Paris 7 and the Hokkaido University.

### 4.2 Whole-cell patch clamp recording

The slice preparation and the method of whole-cell patch clamp recording are described in the Supplementary Methods. Briefly, the cells were voltage clamped using a whole-cell clamp configuration. The parvocellular cells were identified by their apparent topographic location and morphology in the PVN in the acute brain slices. After establishing a stable whole-cell clamp configuration, the cells were identified as neurons by evoking action potential by injecting +10 pA current, then TTX was added to the aCSF following the application of either picrotoxin (PTX, 100 μM) in the case of mEPSC recording, or kinurenic acid (2 mM) in the case of recording mIPSCs. To test the effect of NPY on mEPSCs and mIPSCs of parvocellular neurons in the PVN, NPY (1 μM) was applied in a single bolus into the recording chamber after recording a 4–5 min control period. For recordings in which the changes in intracellular calcium levels were blocked, EGTA was substituted with BAPTA (10 mM) in the intracellular recording solution. In further experiments, inhibitors were dissolved in the aCSF already containing TTX and PTX or kinurenic acid. These drugs were as follows: CB1-antagonist AM251 (1 or 4 μM), non-selective nitric oxide synthase (NOS) inhibitor l-NAME (L-NG-Nitroarginine Methyl Ester, 100 μM), and selective neuronal NOS (nNOS) inhibitor NPLA (N^ω^-propyl-l-arginine, 100 nM).

### 4.3 Intracellular calcium imaging

Parvocellular neurons of the PVN (N = 9) were patched as described above and individually loaded with OGB-1 (20 μM; Invitrogen) Ca^2+^-indicator fluorescent dye *via* whole-cell patch clamp configuration. The experimental conditions in the case of each recorded cells are as follows: firing protocol to test the viability of neurons, NPY treatment (1 μM) in the presence of TTX (600 nM) followed by glutamate (100 μM) treatment to test again the viability of neurons.

To test the mechanisms of the NPY induced increase of the intracellular Ca^2+^ levels, after the firing protocol, the sections were pretreated with TTX combined either with a PLCβ inhibitor, U73122 (5 μM), or a specific ryanodine receptor inhibitor, Dantrolene (5 μM). These pretreatments lasted at least 15–20 min before the recording started. Every treatment period was preceded with a control period of recording. Both Ca^2+^-imaging and electrophysiological data were analyzed offline. Neurons that did not show increase of OGB-1 fluorescence intensity during firing or after glutamate treatment were excluded from the analyses. The experimental procedure is described in details in the Supplementary Methods.

### 4.4 Quadruple-labeling immunofluorescence of the elements of the endocannabinoid and NO signaling systems and markers of glutamatergic and GABAergic neurons

Under deep pentobarbital anesthesia (100 mg/kg of body weight, i.p.), mice were perfused transcardially with 4% paraformaldehyde in 0.1 M phosphate buffer (PB, pH 7.2) for 10 min. Sections (50 μm in thickness) were prepared on a Vibratome (VT1000S; Leica) and incubated with 10% normal donkey serum for 20 min, followed by incubation in the mixture of primary antibodies overnight (1 μg/ml), and then in a mixture of fluorochrome-conjugated species-specific secondary antibodies for 2 h (1:200; Life Technologies; Jackson ImmunoResearch; Abcam). The following primary antibodies were used: rabbit anti-CB1 [44], rabbit anti- DAGLα [45], mouse anti-MAP2 (Millipore; AB15452), guinea pig anti-nNOS [46], goat anti-VGLUT1 [47], goat anti-VGLUT2 [47], and goat anti-VIAAT [47] antibodies. PBS containing 0.1% Tween20 was used as dilution and washing buffer.

### 4.5 Single-labeling immuno-electron microscopy using silver intensified colloidal gold particles

Tissue preparation is described in the Supplementary Methods. Pretreated sections were incubated in rabbit anti-nNOS serum (1:200 Zymed Laboratories) for 4 days at 4 °C, rinsed in PBS and in 0.1% cold water fish gelatin/1% bovine serum albumin (BSA) in PBS, incubated in donkey anti-rabbit IgG conjugated with 0.8 nm colloidal gold (Electron Microscopy Sciences, Fort Washington, PA) diluted at 1:100 in PBS containing 0.1% cold water fish gelatin and 1% BSA for 1 h. After washing, the sections were fixed in 1.25% glutaraldehyde in 0.1 M PB for 10 min. The gold particles were silver intensified with the Aurion R-Gent SE-LM Kit (Aurion) after rinsing in 0.2 M sodium citrate (pH 7.5).

### 4.6 Single-labeling immuno-electron microscopy using nickel-diaminobenzidine chromogen

Pretreated sections were placed in rabbit anti-sGCα1 serum (1:4000) diluted in 2% normal horse serum (NHS) in PBS for 4 days at 4 °C. After rinsing in PBS, the sections were incubated in biotinylated donkey anti-rabbit IgG diluted at 1:500 (Jackson ImmunoResearch Laboratories) in PBS containing 2% normal horse serum (NHS). After rinsing in PBS and treatment with avidin–biotin–peroxidase complex (ABC Elite 1:1000), the sGCα1-immunoreactivity was detected in 0.05% DAB/0.15% Ni-ammonium-sulfate/0.005% H_2_O_2_ in 0.05 M Tris buffer (pH 7.6).

### 4.7 Double-labeling immuno-electron microscopy

Pretreated sections were placed in a mixture of rabbit anti-nNOS serum (1:200) and sheep anti-CB1 serum (1:800) for 4 days at 4 °C. After rinsing in PBS and 0.1% cold water fish gelatin/1% bovine serum albumin (BSA) in PBS, they were incubated in a cocktail of donkey anti-rabbit IgG conjugated (1:100, Science Services, Germany) with 0.8 nm colloidal gold (Electron Microscopy Sciences, Fort Washington, PA) diluted at 1:100 and biotinylated donkey anti-sheep IgG diluted at 1:500 in PBS containing 0.1% cold water fish gelatin and 1% BSA. After washing, the sections were fixed in 1.25% glutaraldehyde in 0.1 M PB for 10 min. The gold particles were silver intensified with the Aurion R-Gent SE-LM Kit (Aurion) after rinsing in 0.2 M sodium citrate (pH 7.5), followed by treatment in avidin–biotin–peroxidase complex (ABC Elite 1:1000). The CB1-immunoreactivity was detected in 0.05% DAB/0.15% Ni-ammonium-sulfate/0.005% H_2_O_2_ in 0.05 M Tris buffer (pH 7.6). The sections were embedded in Durcupan ACM epoxy resin and studied with JEOL-100 C transmission electron microscope. The detailed method of the embedding and ultrastructural examination of the immunostained sections is described in the Supplementary Methods.

### 4.8 Implantation of bilateral guide cannula in the PVN of mice

A bilateral 26-gauge, 0.8 mm C/C stainless steel cannula (Plastics One, Roanoke, Va, USA) was stereotaxically implanted into the PVN under isoflurane anesthesia −0.8 mm posterior to bregma and to a depth of −4.8 mm from the surface of the brain, with bregma and lambda kept in the horizontal plane. The cannula was secured to the skull using dental acrylic cement, then occluded with a dummy cannula. After a week recovery and demonstration that no loss of body weight was observed, correct placement of the cannula was verified by administration of neuropeptide Y (0.25 nM/μl at 0.5 μl/min) using a 33-gauge stainless internal injector cannula.

### 4.9 IntraPVN infusions in mice

Peptides and reagents were dissolved in artificial cerebrospinal fluid (aCSF, prepared according to the instructions of Alzet, Cupertino, CA) and infused intraPVN (0.4 μl on each side) at a rate of 0.5 μl/min. Briefly, the bilateral internal cannula was connected to a polyethylene −50 tubing (Alzet, Cupertino, CA), the tubing connected to a 10 μl Hamilton syringe (Hamilton, Reno, NV) and the syringe was driven by a micro pump (KDS scientific, Fr). Once the internal injector cannula was in place, the animals were set loose during the infusion (1 min), and the injector cannula maintained in place for another minute before replacing the animals in their cages. In experiments in which both CB1 antagonist or NOS inhibitor was administered before NPY, at first the antagonist/inhibitor was administered into the PVN, the internal cannula was replaced and 10 min later, NPY was administered into the PVN using the same method.

All animals were acclimated inside the calorimetric cage (TSE system, Germany) 48 h before the experiment. Animals were kept under a 12 h light/dark cycle from 7.00 am and housed in a temperature-controlled environment at 22.5 °C with free access to food and drink. Animals were customized daily to manipulation.

Body mass composition (lean tissue mass, fat mass, free water, and total water content) was analyzed using an Echo Medical systems' EchoMRI (Whole Body Composition Analyzers, EchoMRI, Houston, USA), according to manufacturer's instructions. Briefly, un-anesthetized mice were weighed before they were put in a mouse holder and inserted in MR analyzer. Readings of body composition were given within 3 min. On the day of the metabolic measurements, at 9 am, appropriate intraPVN injections were performed within 4 min (handling and injection), and the animals placed back into the calorimetric cage in the presence (*ad libitum*) or absence of food. In the latest, food was restored to the food restricted animals 7 h after the intraPVN injections.

### 4.10 Measurement of metabolic parameters

In a first series of study, food of animals was removed 30 min before injection to prevent the confounding effects of the consumed food when the effect of intraPVN injections were investigated on parameters of energy homeostasis such as energy expenditure, estimation of basal metabolism, substrate utilization (RER) and locomotor activity. Animal information, indirect calorimetry, activity measurements, and reagents are described in the Supplementary Methods. Reagent injection days were alternated with aCSF injection. (i.e.: day 1: aCSF, day 2: NPY, day 3 and 4: aCSF, day 5: NPY + AM251, day 6 and 7: aCSF, day 8: AM251).

In a second, similar injection paradigm, food intake and spontaneous locomotor following intraPVN injections was investigated. Animals were placed in their cages with free access to regular chow. Food consumption, metabolic parameters, and locomotor activities were recorded over 8 h. Finally, caloric response, energy homeostasis and locomotor activity were investigated in animals subject to intraPVN injections of either aCSF or NPLA after an overnight fast. Food being replaced 15 min after the initial intraPVN injection.

### 4.11 Statistics

#### 4.11.1 Statistical analysis of electrophysiological recordings

Event detection was performed using the Clampfit module of the PClamp 9.2 and 10.4 software (Molecular Devices). All data for each experiment were normalized relative to baseline and described as mean ± standard error of mean (SEM). Measured parameters of the miniature potentials were as follows: peak amplitude (pA), event frequency (Hz), time to peak (ms), half-width (ms), rise time 10–90 (ms), and rise tau (ms). Data were analyzed using the paired-Student's t-test in the case of self-controlled experiments (before and after NPY treatment). One-way analysis of variance (ANOVA) followed by the Student-Newman-Keuls (SNK) test was used to compare NPY effects among different treatment groups. An α-level of P < 0.05 was regarded as statistically significant in all statistical tests.

The calcium imaging data were compared by Student's t-test.

#### 4.11.2 Statistical analysis of the *in vivo* data

The results are expressed as mean ± SEM. Variance equality was analyzed by F test (Microsoft Excel), and comparisons between groups were carried out using Student's t test or nonparametric Mann-Whitney-Wilcoxon's test (Minitab, Paris, France). When appropriate, analyses of variances were performed followed either by a Bonferroni post hoc test with the appropriate parameters (drugs and time), and their interaction as factor (Minitab, Paris, France) a, b, c and d differed significantly (P < 0.05). ANCOVA analyses with the locomotor activity as covariate was used to determine the effect of the increased locomotor activity on the energy expenditure in the first 2.5 h after drug administration. Unless otherwise indicated in the text, a P-value of <0.05 was indicated by × and considered statistically significant. Data in the line figures are expressed as means ± SEM. Bar graphs represent means ± SEM obtained from ANOVA. For each phase, values (bars) without the same labels (a, b, c and d) differ significantly.
