# Supplementary material for: Endocannabinoid and nitric oxide systems of the hypothalamic paraventricular nucleus mediate effects of NPY on energy expenditure
Source: Mol Metab. 2018 Sep 18;18:120–33. doi: 10.1016/j.molmet.2018.08.007 (PMC6308028; doi:10.1016/j.molmet.2018.08.007)
Supplement: Multimedia component 1 [file mmc1.docx]

**Supplementary Methods**

*Slice preparation for electrophysiological recordings*

Mice were deeply anesthetized with isoflurane and subsequently decapitated. Brains were rapidly removed from the skull and placed into ice-cold cutting solution saturated with 95% O_2_ and 5% CO_2_ (carbogen gas). The cutting solution of slices prepared for the measurement of miniature excitatory postsynaptic currents (mEPSCs) was prepared as follows: (in mM): 140 NaCl, 3KCl, 1.3 MgSO_4_, 1.4 NaH_2_PO_4_, 2.4 CaCl_2_, 11 glucose, 5 HEPES, pH 7.25, 280–290 mOsm/L); while the cutting solution of the slices prepared for the mIPSC recordings had the following composition: (in mM): 87 NaCl, 2.5 KCl, 7 MgCl_2_, 26 NaH_2_PO_4_, 0.5 CaCl_2_, 25 glucose, 75 sucrose, (pH 7.4; 280–290 mOsm/L). Hypothalamic blocks were dissected from the mouse brains and 250-μm-thick coronal slices were cut using VT1000S or VT1200S vibratome (Leica).

The slices were bisected along the third ventricle and transferred into a holding chamber containing artificial CSF (aCSF) with the following composition: (in mM): 140 NaCl, 3 KCl, 1.4 NaH_2_PO_4_, 2 MgCl_2_, 2.4 CaCl_2_, 26 NaHCO_3_, and 11 glucose, saturated with carbogen gas, (pH 7.4; 280–290 mOsm/L) at room temperature for at least 1.5 h before the recording.

Slices were transferred to a submersion type of recording chamber. In the case of the mEPSC recordings to block GABA_A_ receptor-mediated IPSCs, picrotoxin (100 µM, Sigma) was included in the aCSF and the solution contained 660 mM tetrodotoxin (TTX). For mIPSC recording, the aCSF contained kinurenic acid (2 mM) and 660 nM TTX. The recordings were performed at 32-33 ^o^C and the flow rate was between 1.5-2 ml/min.

*Whole-cell patch clamp recording*

The cells were voltage clamped using a whole-cell clamp configuration as follows: cells were visualized with an upright microscope (Eclipse FN-1; Nikon) with infrared Nomarski differential interference contrast optics and a CCD camera (Zyla; ANDOR). Whole-cell patch-clamp recordings were made using Multiclamp 700B amplifiers (Molecular Devices) with pClamp10.4 software. Data were filtered at 3 kHz using the built-in Bessel filter of the amplifier and digitized at 15 kHz.

The resistance of the patch electrodes was 2-5 MΩ. The intracellular pipette solution used for mEPSC electrophysiological recording contained (in mM) 10 HEPES, 120 K-gluconate, 10 KCl, 1 NaCl, 1 MgCl_2_, 1 EGTA, 2 Mg-ATP, 0.3 Na-GTP, and 2 biocytin (pH 7.25; 290-295mOsm/L. For mIPSC recording, the intracellular solution contained the following (in mM): 130 CsCl, 8 NaCl, 0.1 CaCl_2_, 0.1 EGTA, 10 HEPES, 4 Mg-ATP, 0.3 Na_2_-GTP, 5 Lidocaine N-ethyl bromide, and 2 biocytin (pH 7.25; 280–300 mOsm/L). For voltage-clamp recordings in both experiments, the holding current was set at -70 mV and series resistance was monitored throughout each experiment. Neurons with a change in series resistance of >25% were excluded from the analysis. Unless otherwise stated, the chemicals for the intracellular and extracellular solutions were purchased from Sigma-Aldrich, inhibitors and antagonists were obtained from Tocris Bioscience.

The parvocellular cells were identified by their apparent topographic location and morphology in the PVN in the acute brain slices. After establishing a stable whole-cell clamp configuration, the cells were identified as neurons by evoking action potential by injecting +10 pA current, then TTX was added to the aCSF following the application of either picrotoxin (100 μM) in the case of mEPSC recording, or kinurenic acid (2 mM) in the case of recording mIPSCs. Equilibration of sections lasted at least 10-15 min before the beginning of the recording.

To test the effect of NPY on mEPSCs and mIPSCs of parvocellular neurons in the PVN, NPY (human-rat; Bachem, 1 μM) was applied after recording a 4-5 min control period. For recordings where the changes in intracellular calcium levels were blocked, EGTA was substituted with BAPTA (10 mM) in the intracellular recording solution. In further experiments, other drugs were dissolved in the aCSF already containing TTX and PTX or kinurenic acid. These drugs were as follows: CB1-antagonist AM251 (1 or 4 μM), non-selective nitric oxide synthase (NOS) inhibitor, L-NAME (L-NG-Nitroarginine Methyl Ester, 100 μM), and selective neuronal NOS (nNOS) inhibitor, NPLA (N^ω^-propyl-L-arginine, 100 nM).

*Intracellular calcium imaging*

The green fluorescent calcium-indicator Oregon Green BAPTA-1 (OGB-1; 20 μM; Invitrogen) was added to the intracellular solution (same as mentioned for the patch clamp electrophysiology, however, without containing EGTA). After establishing whole-cell configuration in parvocellular neurons in the PVN, dye was allowed to diffuse into the perikaryon for at least 40 min before starting calcium imaging.

Andor AMH-200-FS6 halide light source was used to illuminate the sections. Light intensity was filtered at an excitation wavelength of 473-491 nm and an emission wavelength of 503-548 nm. Images were collected at a 1 Hz sampling rate with 100 ms exposure time using an Andor Zyla 5.5 camera (Andor Technology, UK) equipped with an optical sectioning device (DSD2, Andor Technology, UK) to generate confocal-like images. All image data were collected and analyzed using Nikon control and analysis software (NIS-Elements AR 4,40 64 bit versions).

Neurons that did not show increase of OGB-1 fluorescence intensity after firing or glutamate treatment were excluded from the analyses. Data were compared by Student’s t-test.

All recordings started after establishing a stabile electrophysiological connection and an unchanging calcium signal. Neurons first were recorded in a control period then a short firing (5-10 sec) period as a stimulus to artificially induce calcium signal in the soma. Then TTX (600 nM) was added to the aCSF, and another control period was recorded that followed by the NPY (1 μM) treatment, following a washout phase another control period was recorded and then sections were treated with glutamate (100 μM). To test the mechanisms of the NPY induced increase of the intracellular Ca^2+^ levels, after the firing protocol, the sections were pretreated with TTX combined either with a PLCβ inhibitor, U73122 (5 μM), or a specific ryanodine receptor inhibitor, Dantrolene (5 μM). These pretreatments lasted at least 15-20 min before the recording started. The firing protocol and the glutamate (100 μM) treatments served as controls, those cells that did not respond to both of these treatments with increased calcium signal intensity were excluded from further analysis. Both electrophysiological data and calcium imaging data recordings were started at the same time, and were analyzed (N=9) offline.

Calcium-imaging analysis was performed by choosing a region of interest (ROI) in the soma not including parts of the patch pipette and the nucleus of the neurons, while another ROI was selected outside of any part of the filled neuron to determine the background fluorescent intensity. The background intensity was subtracted from the fluorescent intensity (in artificial units) of the soma; these values were compared between the control and treatment conditions. Every treatment period, mentioned above has its own control period, recorded just before the treatment period and treatment intensities were determined as percentages of its own control periods.

*Tissue preparation for light microscopic immunocytochemistry*

Six mice were used for the morphological studies. For light microscopy, the animals were deeply anesthetized with ketamine/xylazine (ketamine 50 mg/kg, xylazine 10 mg/kg body weight, ip), and perfused transcardially for 3 min with 0.01 M phosphate-buffered saline (PBS), pH 7.4, followed sequentially by perfusion with 4% paraformaldehyde in 0.1 phosphate buffered (PB), pH 7.4, for 10 min and then with 10% sucrose in 0.01 M PBS for 3 min. The brains were rapidly removed and stored in 20% sucrose in PBS for 2 h at room temperature. For electron microscopy, after identical anesthesia, the animals were perfused transcardially with 10 ml 0.01 M phosphate-buffered saline (PBS), pH 7.4, followed sequentially by 10 ml of 4% paraformaldehyde in Na-acetate buffer, pH 6.0, and then by 50 ml of 4% paraformaldehyde in Borax buffer, pH 8.5. The brains were rapidly removed and stored in 4% paraformaldehyde in 0.1 M phosphate buffer (PB), pH 7.4, for 24 h at 4 °C.

*Tissue preparation for ultrastructural studies*

Serial 25 µm thick coronal sections were cut on a Leica VT 1000S vibratome (Leica Microsystems, Wetzlar, Germany) through the PVN. The sections were treated with 0.5% H_2_O_2_ in PBS for 15 min., cryoprotected in 15% sucrose in PBS for 15 min at room temperature and in 30% sucrose in PBS overnight at 4°C, and then quickly frozen over liquid nitrogen and thawed three times to improve antibody penetration into the tissue. The sections were then processed for single- and double-labeling immunocytochemistry as described below.

*Embedding and ultrastructural examination of the immunostained sections*

After immunocytochemistry, the sections were osmicated for 1h at RT, and then treated with 2% uranyl acetate in 70% ethanol for 30 min. Following dehydration in an ascending series of ethanol and acetonitrile (Sigma), the sections were flat embedded in Durcupan ACM epoxy resin (Fluka) on liquid release agent (Electron Microscopy Sciences)-coated slides, and polymerized at 56 °C for 2 days. After polymerization, 60–70 nm thick ultrathin sections were cut with Leica ultracut UCT ultramicrotome (Leica Microsystems, Wetzlar, Germany). The ultrathin sections were mounted onto Formvar-coated, single slot grids, contrasted with 2% lead citrate (Sigma Aldrich, Hungary) and examined with a JEOL-100 C transmission electron microscope.

*Specificity of antisera*

The specificity of nNOS, sGCα1, CB1, NPY, VGLUT2 and VIAAT antibodies were reported previously [S1, S2, S3, S4, S5, S6]. CB1 immunoreactivity was completely absent from hypothalamic sections of the CB1-KO mice.

*Animals, indirect calorimetry and activity measurements using beam breaks*

Adults male mice CD1 (25–30 g, Janvier, Le Genest Saint Isle, Fr) were housed in stainless steel cages in a room maintained at 22.5 ± 1°C with light from 7:00 am. to 7:00 pm. Food (#A03, Safe, Augy, France) and water were given *ad libitum* unless otherwise stated. Mice were kept individually for at least 7 days in their own metabolic cages with training bottle and training food container (TSE system) before the cage (bedding included) being placed in the calorimetric platform. All animals were acclimated for 48 h in calorimetric cages before experimental measurements.

Mice were analyzed for whole energy expenditure (EE), oxygen consumption and carbon dioxide production, respiratory exchange rate (RER = VCO_2_/VO_2_, where V is volume), and locomotor activity using calorimetric cages with bedding, water and with or without food (Labmaster, TSE Systems GmbH, Bad Homburg, Germany). The ratio of gases was determined through an indirect open circuit calorimeter [S7, S8]. This system monitors O_2_ and CO_2_ concentration by volume at the inlet ports of a tide cage through which a known flow of air is being ventilated (0.4 L/min), and compared regularly to a reference empty cage. The air sensors were calibrated with O_2_ and CO_2_ mixture of known concentrations (Air Liquide, S.A. France). Oxygen consumption, carbon dioxide production and EE were recorded every 10 min for each animal during the entire experiment. Whole energy expenditure was calculated using the Weir equation respiratory gas exchange measurements [S9]. Ambulatory movement was recorded using an infrared light beam-based activity monitoring system with online measurement at 100 Hz.

Data analysis was performed using Excel XP using extracted raw value of VO_2_ consumed, VCO_2_ production (expressed in ml/h), and energy expenditure (Kcal/h). Subsequently, each value was normalized to whole lean tissue mass extracted from the EchoMRI analysis.

*Estimation of basal metabolism*

No generally accepted methods of estimation of the basal metabolism are available presently [S10]. An estimation of basal metabolism was calculated from the first experimental set when the animals did not have access to food to avoid confounding thermic effects of food. Data points of EE were considered to be the best estimation of basal metabolism when spontaneous activity during the previous 30 minutes was less than 1% of the highest daily value.

*Reagents*

Animals removed from the calorimetric cages (TSE system) were weighted and injected either with aCSF, 0.25 nM NPY alone (Tocris, Lille, France.) or 0.25 nM NPY combined with either 40 pM of a potent CB1 antagonist (Tocris, Lille, France.) or 0.8 nM of a potent inhibitor of nNOS (Tocris, Lille, France. ).

**Supplemental references**

[S1] Miura, E., Fukaya, M., Sato, T., Sugihara, K., Asano, M., Yoshioka, K., and Watanabe, M.J. (2006) E xpression and distribution of JNK/SAPK-associated scaffold protein JSAP1 in developing and adult mouse brain. Neurochemistry. 97, 1431-1446.

[S2]. Szabadits, E., Cserép, C., Ludányi, A., Katona, I., Gracia-Llanes, J., Freund, T.F., and Nyíri, G. (2007) Hippocampal GABAergic synapses possess the molecular machinery for retrograde nitric oxide signaling. The Journal of Neuroscience 27, 8101-8111.

[S3] Sardella, T.C., Polgar, E., Watanabe, M., and Todd, A.J. (2011). A quantitative study of neuronal nitric oxide synthase expression in laminae I-III of the rat spinal dorsal horn. Neuroscience *192*, 708-720.

[S4] Szabadits, E., Cserep, C., Szonyi, A., Fukazawa, Y., Shigemoto, R., Watanabe, M., Itohara, S., Freund, T.F., and Nyiri, G. (2011). NMDA receptors in hippocampal GABAergic synapses and their role in nitric oxide signaling. The Journal of Neuroscience *31*, 5893-5904.

[S5] Wittmann, G., Liposits, Z., Lechan, R.M., and Fekete, C. (2002). Medullary adrenergic neurons contribute to the neuropeptide Y-ergic innervation of hypophysiotropic thyrotropin-releasing hormone-synthesizing neurons in the rat. Neuroscience letters *324*, 69-73.

[S6] Fukudome, Y., Ohno-Shosaku, T., Matsui, M., Omori, Y., Fukaya, M., Tsubokawa, H., Taketo, MM., Watanabe, M., Manabe, T., and Kano, M. (2004). Two distinct classes of muscarinic action on hippocampal inhibitory synapses: M2-mediated direct suppression and M1/M3-mediated indirect suppression through endocannabinoid signalling. European Journal of Neuroscience 19, 2682-2692

[S7] Arch, J.R., Hislop, D., Wang, S.J., and Speakman, J.R. (2006). Some mathematical and technical issues in the measurement and interpretation of open-circuit indirect calorimetry in small animals. International Journal of Obesity 30, 1322-1331.

[S8] Even, P.C., Mokhtarian, A., and Pele, A. (1994). Practical aspects of indirect calorimetry in laboratory animals. Neuroscience and Biobehavioral Reviews 18, 435-447.

[S9] Weir, J.B. (1949). New methods for calculating metabolic rate with special reference to protein metabolism. The Journal of Physiology *109*, 1-9.

[S10] Even, P.C., and Nadkarni, N.A. (2012). Indirect calorimetry in laboratory mice and rats: principles, practical considerations, interpretation and perspectives. American Journal of Physiology. Regulatory, Integrative and Comparative Physiology 303, R459-476.

Supplementary Figures

**
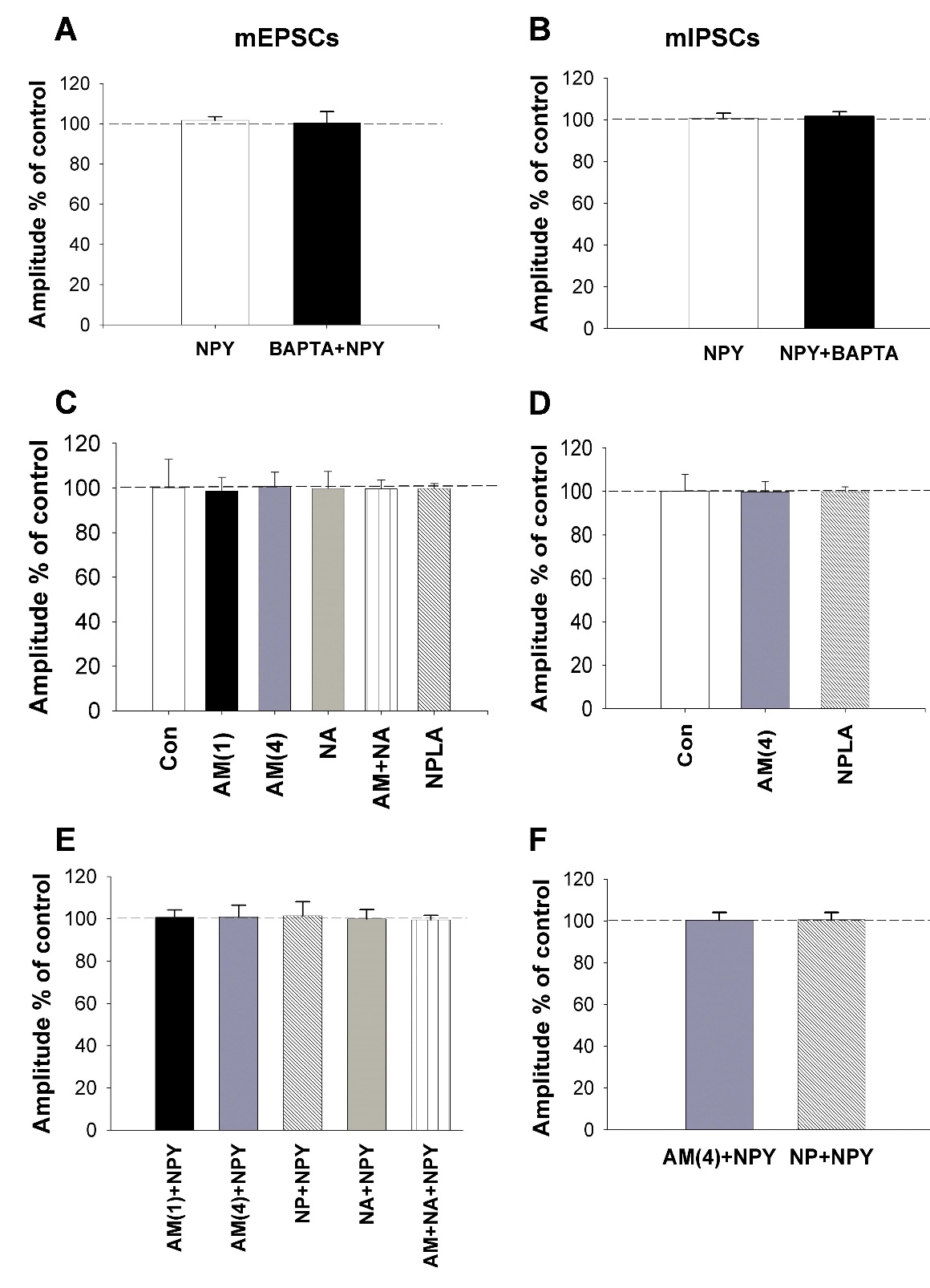
**

**Supplementary Figure 1. The used treatments had no effect on the amplitude of mEPSCs and mIPSC of the parvocellular neurons in the PVN.** Bar graphs (A, C, E) show the effect of different treatments on the amplitude of mEPSCs. Bar graphs (B, D, F) show the effect of different treatments on the amplitude of mIPSCs. No significant change is detected in either treatment group. Abbreviations: AM(1): 1 μM AM251 treatment; AM(4): 4 μM AM251 treatment; NP: NPLA treatment (100 nM); NA: L-NAME treatment (100 μM); AM+NA: 1 μM AM251 and L-NAME (100 μM) co-treatment.


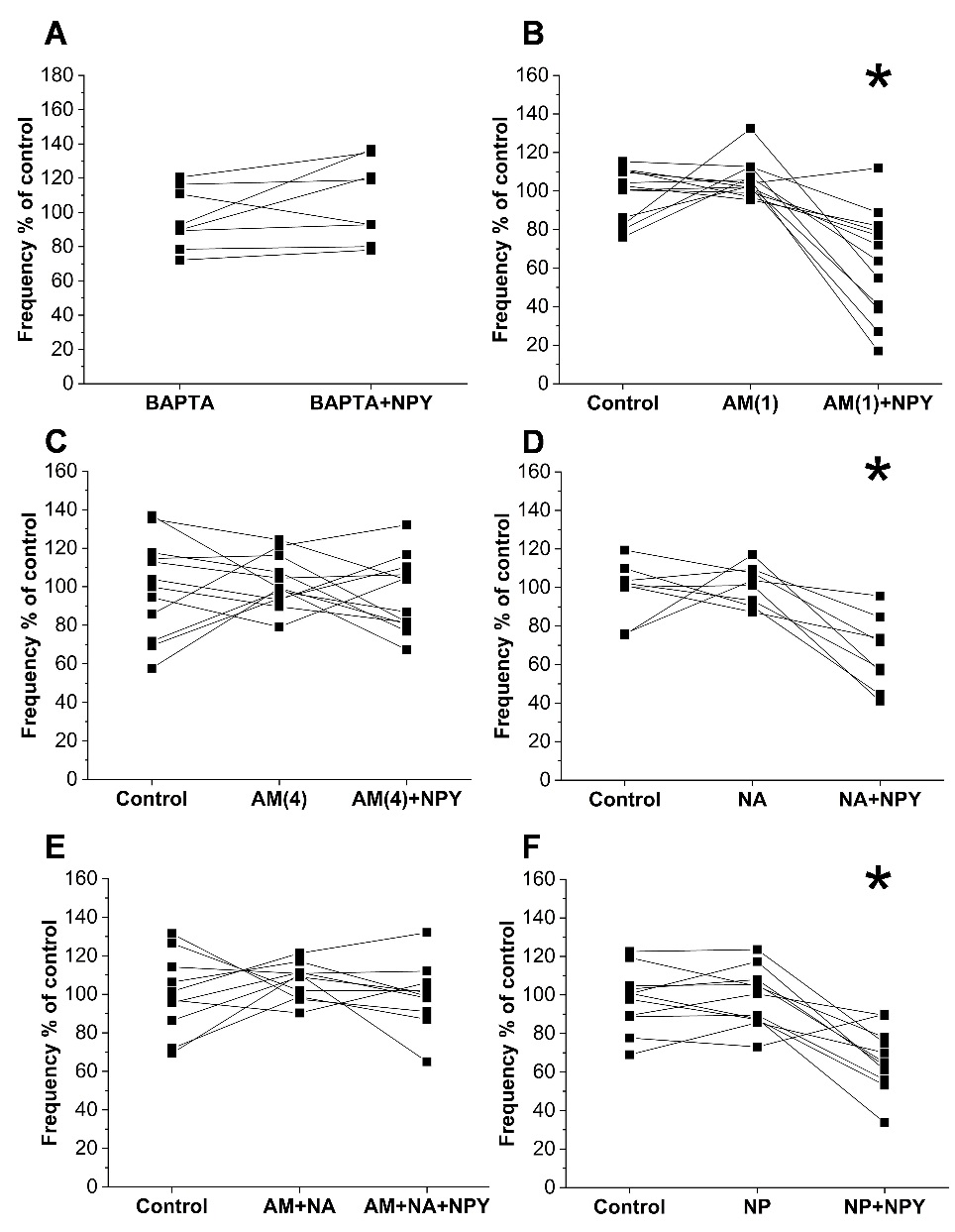


**Supplementary Figure 2. Related to figure 4. Effects of BAPTA, AM251, L-NAME, NPLA and combined AM251+L-NAME treatments on the effect of NPY on the mEPSC frequency of parvocellular PVN neurons.** Every measured parvocellular neuron is represented as line connected dots in different experimental conditions such as, control, antagonist only treatment, and antagonist+ NPY treatment. Intracellularly applied BAPTA (10 mM) completely abolished the effects of NPY on the mEPSCs frequency (N_cells_=8; N _mice_=6) (A). Low dose (1 μM) of CB1 receptor antagonist AM251 (B) failed to influence (P< 0.001, T=4.811) the effect of NPY (1 μM; N_cells_=12; N_mice_=6). Higher dose (4 μM) of AM251 (C) blocked the NPY-induced inhibition of mEPSCs frequencies (N_cells_=12; N_mice_=4). Non-selective NOS inhibitor L-NAME (D) (100 μM; N_cells_=8; N_mice_=6) did not influence (P<0.001, T= 6.494) the effect of NPY on the mEPSC frequencies. Combination of low dose (1 μM) of AM251 and L-NAME (E), however, abolished the effect of NPY on frequencies of mEPSCs (N_cells_=10; N_mice_=6). The selective nNOS inhibitor NPLA (100 nM; N_cells_=11; N_mice_=7) did not alter (P=0.004, T= 3.124) the inhibitory effect of NPY (1 μM) on mEPSCs (F). Abbreviations: AM(1): 1 μM AM251 treatment; AM(4): 4 μM AM251 treatment; NP: NPLA treatment (100 nM); NA: L-NAME treatment (100 μM); AM+NA: 1 μM AM251 and L-NAME (100 μM) co-treatment.


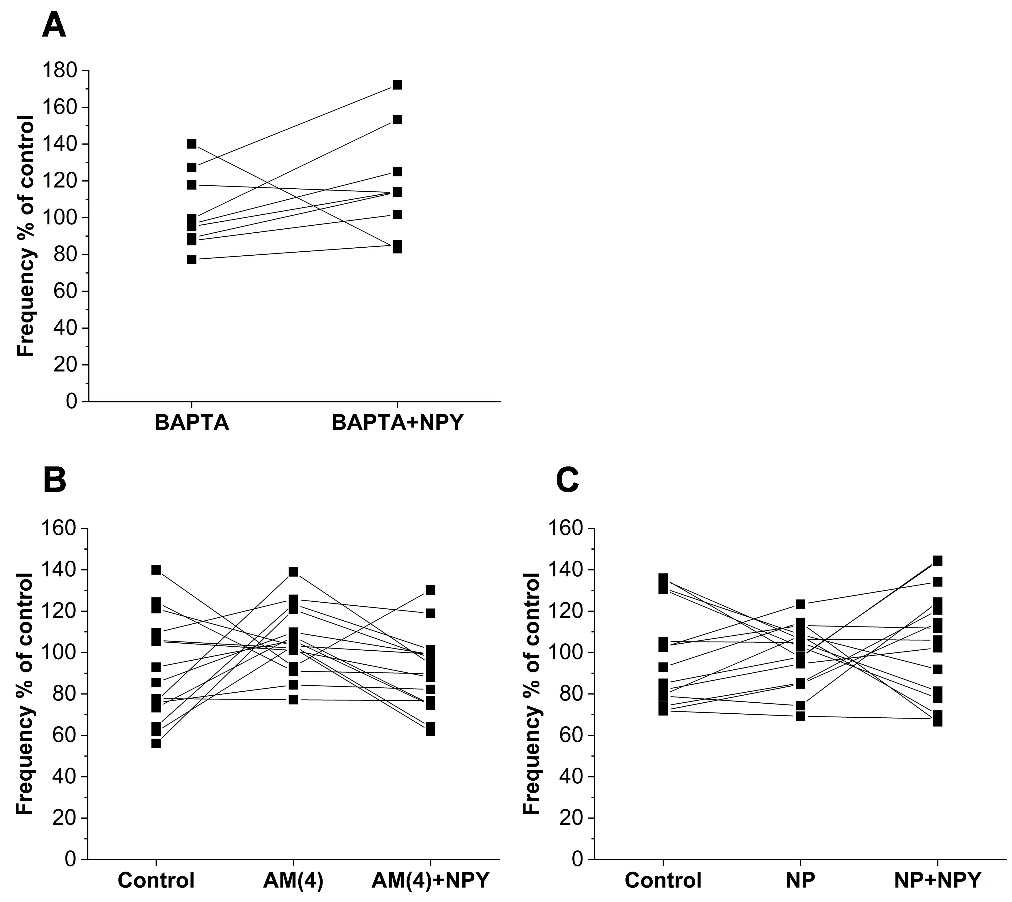


**Supplementary Figure 3. Related to figure 4. Effects of BAPTA, AM251, and NPLA treatments on the effect of NPY on the mIPSC frequency of parvocellular PVN neurons.** Every measured parvocellular neuron is represented as line connected dots in different experimental conditions such as, control, antagonist only treatment, and antagonist+ NPY treatment. Intracellularly applied BAPTA (10 mM) (A) completely abolished effects of NPY on the mIPSCs frequency (N_cells_=9; N_mice_=5). Higher dose (4 μM) AM251 (B) blocked the NPY-induced inhibition of mIPSCs frequencies (N_cells_=15; N_mice_=6). The selective nNOS inhibitor NPLA (100 nM; N_cells_=15; N_mice_=6) abolished the effect of NPY on the event frequencies of mIPSCs (C). Abbreviations: AM(4): 4 μM AM251 treatment; NP: NPLA treatment (100 nM).


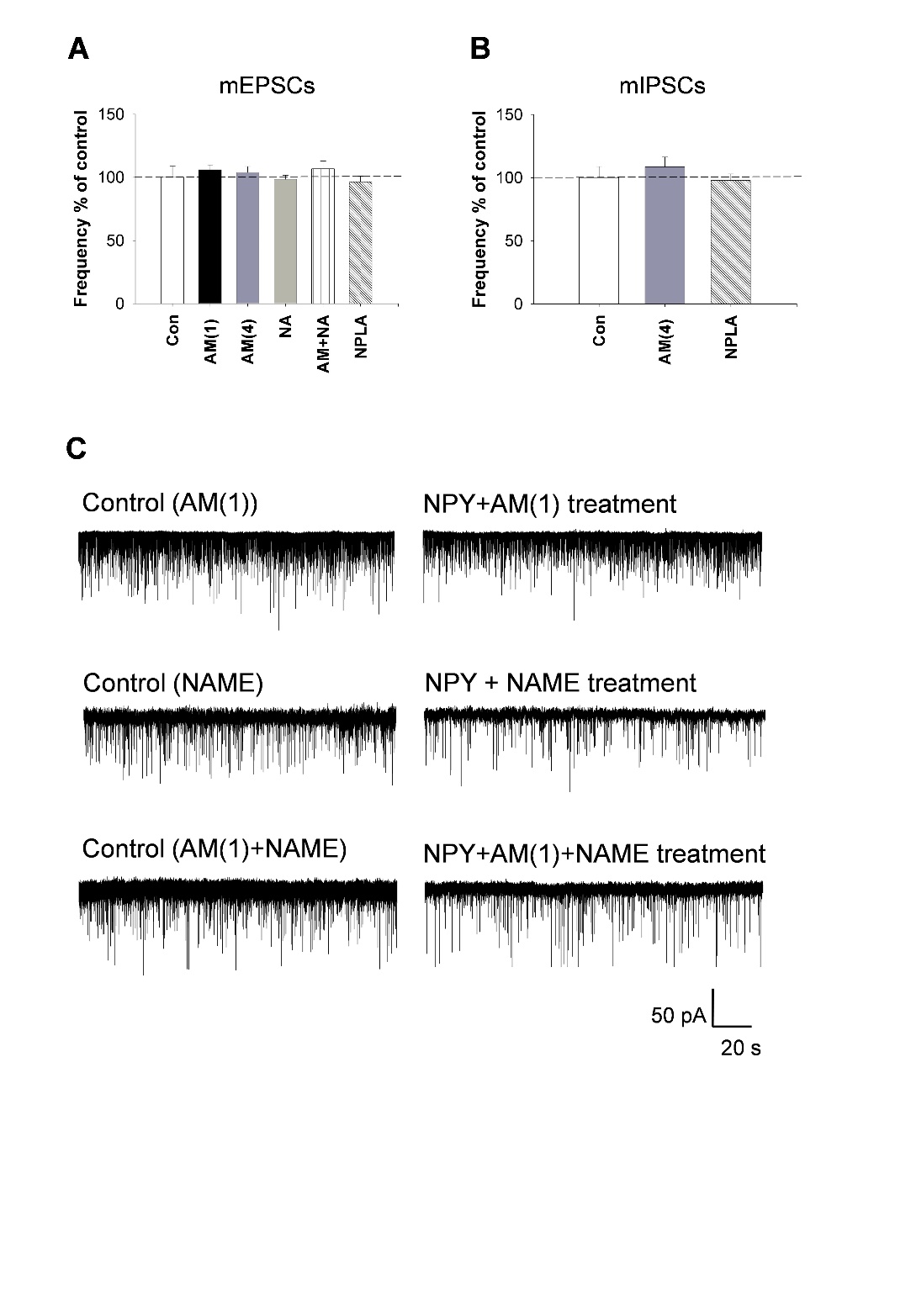


**Supplementary Figure 4. Effects of the CB1 and NOS inhibitors on the inputs of the parvocellular neurons in the PVN.** Bar graphs (A and B) show the effect of different inhibitor treatments without NPY treatment on the frequency of the mEPSCs (A) and mIPSCs (B) of the parvocellular neurons in the PVN. Administration of 1 μM (N_cells_ =12; N_mice_=6)or 4 μM (N_cells_ N=12; N_mice_=4) CB1 receptor antagonist AM251, the non-selective NOS inhibitor L-NAME (N_cells_=8; N_mice_=6) nor the selective nNOS inhibitor NPLA (N_cells_=11; N_mice_=7) had any effect on the frequencies of mEPSCs. The combination of L-NAME and the subthreshold dose of AM251 (1μM) also did not affect significantly the frequencies of mEPSCs (N_cells_=10; N_mice_=6; A). (B) Event frequencies of mIPSCs were not affected by either the 4 μM AM251 treatment (N_cells_=15; N_mice_=6) or NPLA treatment (N_cells_=15; N_mice_=6) when neurons were treated alone with the inhibitors.
Representative whole-cell patch-clamp recordings (C) illustrate the effects of CB1 and nNOS inhibition on the NPY induced regulation of the frequency of mEPSCs of the parvocellular neurons. These data are shown as bar graphs on Fig.4.A. Abbreviations: AM(1): 1 μM AM251 treatment; AM(4): 4 μM AM251 treatment; NP: NPLA treatment; NA: L-NAME treatment; AM+NA: 1 μM AM251 and L-NAME co-treatment.

*
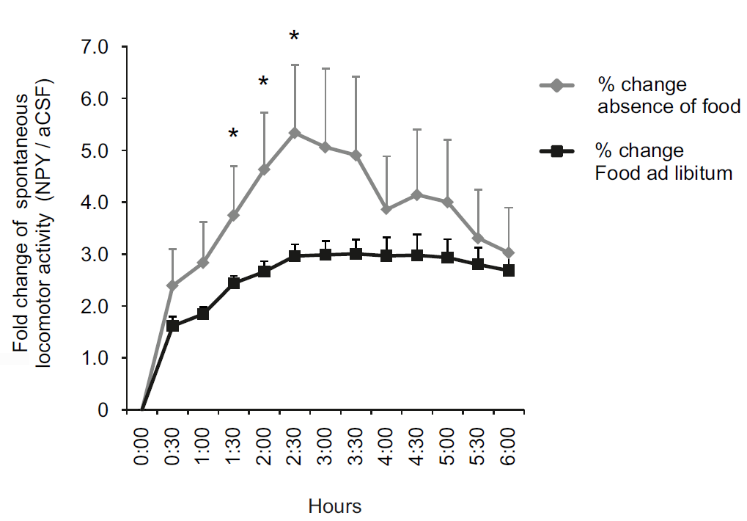
*

**Supplementary Figure 5. Fold change in spontaneous locomotor activity in mice injected intraPVN with NPY in presence or absence of food.** Fold change of spontaneous locomotor activity of mice after intraPVN injection of NPY compared to CSF injected mice in the presence (black) or absence of food (grey) monitored for 6 hours post injection. Note that NPY markedly increases the locomotor activity both in the presence and absence of food, but the NPY induced increase of locomotor activity is significantly higher in the absence of food. Data are expressed as mean ± SEM. * p<0.05. n = 8 per group. Comparisons between groups were carried out using Student’s t test.

*
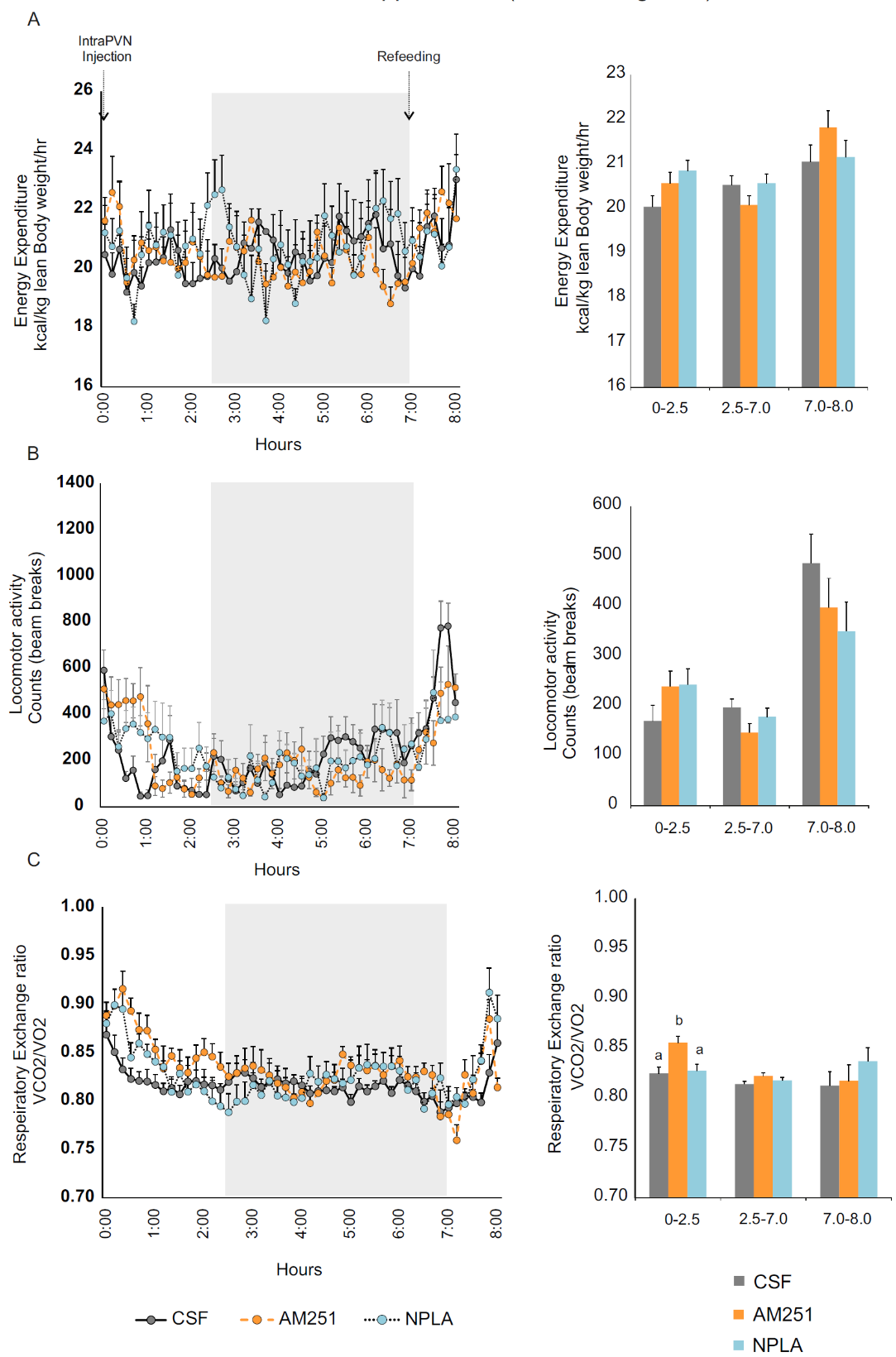
*

**Supplementary Figure 6**. **Related to Figure 5. Effect of intraPVN administration of AM251 or NPLA alone on energy expenditure normalized by total lean body mass (A), spontaneous locomotor activity (B), and respiratory exchange ratio (RER) (C) of mice with no access to food.** AM251 had no effect on the energy expenditure and the locomotor activity and only slightly increased the RER in the first period. NPLA had no effect on any parameters. Each group was injected either with CSF (grey, n=8 mice), AM251 (orange, n=8 mice), or NPLA (light blue, n=7 mice) at T=0, and food was replaced at T=7 h. Bar graphs were calculated from the ANOVA analysis for each phase. Data with different superscript letters are significantly different (P<0.05) according to the ANOVA analysis followed by a Bonferroni post hoc test. Data are expressed as mean ± SEM.

*
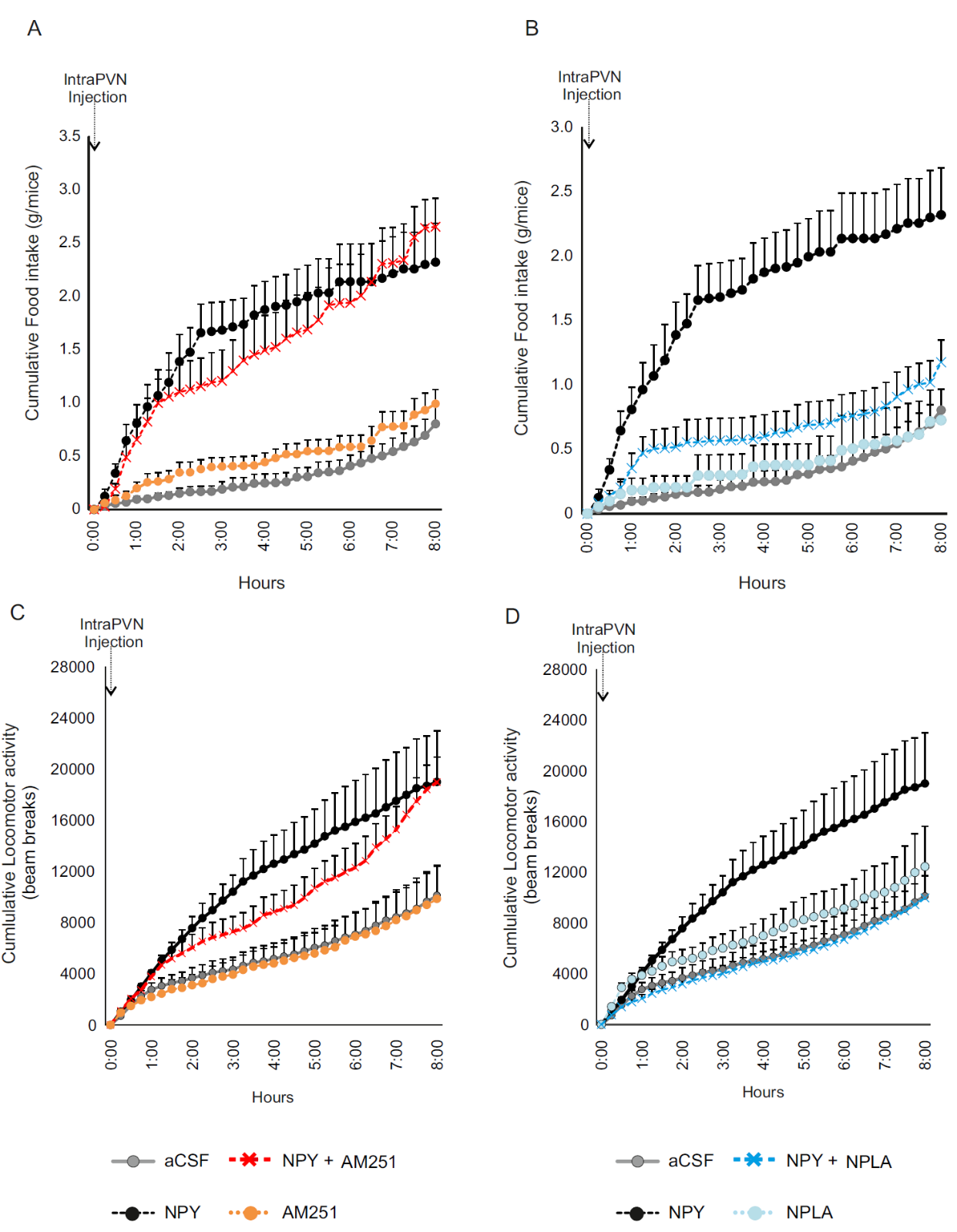
*

**Supplementary Figure 7**. **Related to Figure 6A,B** Cumulative food intake (A,B) and cummulative spontaneous activity (C,D) of mice injected intraPVN with CSF (grey), NPY (black) alone, AM251 alone (orange), NPLA alone (light blue) and co-administrated with AM251+NPY (red) or NPLA+NPY (blue). Note that intraPVN administration of AM251 alone or in combination with NPY had onl minor effect on the food intake (A) and locomotor activity (C). Administration of NPLA alone did not influence the measured parameters (B,D), but NPLA prevented the effects of NPY on both the food intake (B) and the locomotor activity (D). Food intake and spontaneous locomotor activity was monitored over 8 hours post injection. n = 8 per group.

*
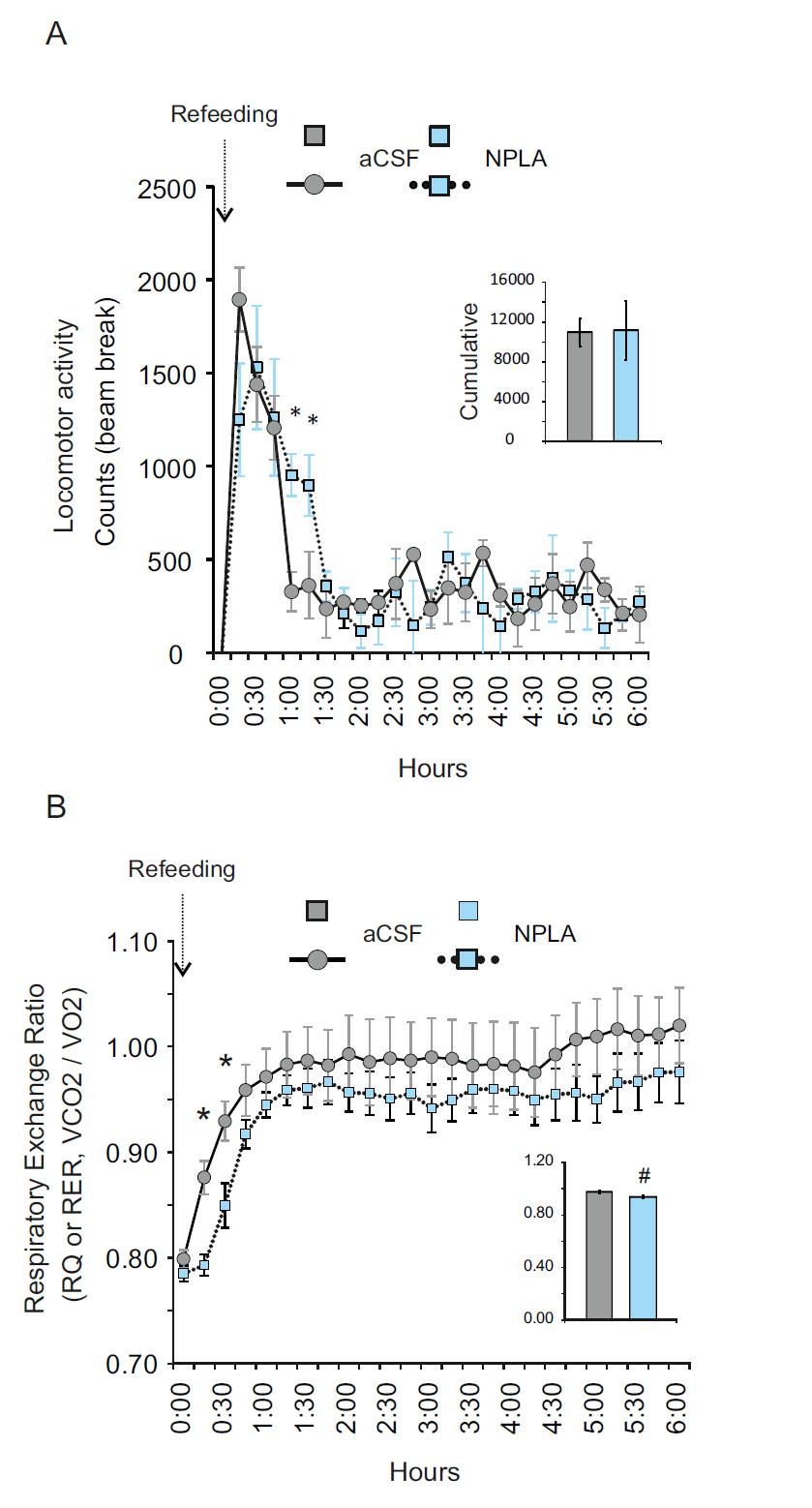
*

**Supplementary Figure 8**. **Related to Figure 6C** **Spontaneous locomotor activity and respiratory exchange ratio after intraPVN injection of NPLA following an overnight fast.** (A) Spontaneous locomotor activity (beam breaks) and (B) respiratory exchange ratio (VCO2/VO2) registered within 6 hours post intra PVN injection of CSF or NPLA. Bar graph in A represents cumulative spontaneous activity. Bar graph in B represents mean ± SEM of ANOVA analysis. * p<0.05, # P<0.001, CSF versus NPLA injected mice. n = 8 per group.
